# A Case-Based Workshop Training Medical Students in Assessing Social Determinants of Health Needs and Connecting With Community Resources

**DOI:** 10.15766/mep_2374-8265.11232

**Published:** 2022-03-21

**Authors:** Kara Burke, Grace Bigham, Gabrielle Ferrara-Leach

**Affiliations:** 1 Director for Community Engagement, Division of Community Outreach and Medical Education, Albany Medical College; 2 Fourth-Year Medical Student, Albany Medical College; 3 Administrative Coordinator, Division of Community Outreach and Medical Education, Albany Medical College

**Keywords:** Social Determinants of Health, Service-Learning, Community-Based Medicine, Diversity, Inclusion, Health Equity

## Abstract

**Introduction:**

Social determinants of health (SDHs) are now recognized as a major factor affecting patient health outcomes and should be addressed by the health care system. Many medical students do not receive practical training on how to assess SDH needs or connect individuals with appropriate resources.

**Methods:**

The authors developed and delivered a 90-minute case-based workshop to train undergraduate medical students in assessing SDH needs and connecting with community resources during their service-learning experiences. The workshop was implemented with undergraduate medical students participating in service-learning programs that linked individuals with resources, with the intention that this information could be used in other educational activities and the students' future medical practice.

**Results:**

Twenty-five students participated in the training. Pre- and postsurveys indicated that the training resulted in statistically significant increases in average knowledge and confidence in all topic areas. All participants reported enhanced knowledge and indicated that they would use this information in service-learning and their future medical practice.

**Discussion:**

In addition to teaching medical students about the impact of SDHs, this cased-based curriculum provides learners with tools to understand the barriers their patients may face as well as how policy and local resources impact them.

## Educational Objectives

By the end of this activity, learners will be able to:

1. Demonstrate how to conduct a basic needs screening on a client or patient.
2. Describe resources, including health insurance resources, available in their community.
3. Describe the basic benefit and eligibility criteria of public insurance programs.
4. Discuss strategies for building trust and communicating effectively with members of marginalized groups.
5. Explain the historical basis for mistrust of the medical system in marginalized communities.

## Introduction

Social determinants of health (SDHs) are categorized as “the non-medical factors that influence health outcomes. They are the conditions in which people are born, grow, work, live, and age, and the wider set of forces and systems shaping the conditions of daily life.”^1^ These determinants create long-standing drivers in health equity and interfere with access to care. The Physicians Foundation's 2018 Survey of America's Physicians found that nearly 90% of U.S. health care providers reported their patients had a serious health problem linked to poverty or other social conditions.^2^ Because of this profound impact on the health and well-being of patients, health care providers and the public health system have a responsibility to address and work to remove SDHs.

In addition to more commonly discussed SDHs, such as food insecurity, housing, and transportation, it is important to acknowledge the role that mistrust of the health care system can play as an SDH and what steps students and health care providers can take to address this mistrust. Mistrust of the health care system functions as a barrier to health care access and medical service use among minority communities.^3^ Mistrust of medicine can be linked both to a troubling history in the United States of medical mistreatment of persons of color and to modern-day instances of institutionalized racism and discrimination towards people of color by health care workers.^3^ Mistrust of the medical system results in underuse of preventative care and screening services, decreased participation in medical research and clinical trials, and withdrawal from care or complete avoidance of medical care or treatment.^4^ Any combination of these factors can contribute to the disparate health outcomes of minority populations. Health care providers and medical students should be trained to understand and recognize that mistrust exists as a barrier to health care use amongst minority patients; health care providers should be able to empathize and communicate with their patients in order to build trust and engage them in a way that promotes their health, well-being, and independent ownership of their health care.^4,5^

The American College of Physicians, the National Academy of Sciences, and the U.S. Department of Health and Human Services all recommend that professionals be taught the effects of social inequality because of its interaction with the health and well-being of an individual.^6–9^ Although there is uncertainty around the best method of incorporating SDHs in medical education, the possible benefits of this education drive medical schools to develop their own programs centered around SDHs. The curriculum can be delivered in a variety of forms, including a single lecture or an expansive service-learning program within the institution.^10–12^ Beyond education, many medical practices, particularly in primary care, have begun to recognize the value of screening for SDHs.^13^ However, there is significant work to be done in creating systems and structures for how to address SDH needs when a patient screens positive. Equipping medical students and, ultimately, health care providers with the knowledge and skills to connect patients with community resources can support them in addressing these needs clinically, either as providers or with their colleagues in social work, nursing, and public health.

We describe a case-based training workshop delivered as part of a service-learning curriculum for undergraduate medical students. Service-learning is an integral part of medical education and a required component for medical school accreditation under Liaison Committee on Medical Education Standard 6.6.^14,15^ Many medical student service-learning programs include activities such as patient navigation and health care system navigation. This work requires students to have functional knowledge about needs assessment, community resources, health insurance, and how to effectively build trust and communicate with various patient populations. This type of service-learning allows students and future providers to understand their patients' experiences with more depth and empathy.^16^

A large part of the current practice and research around SDHs in medical education focuses on teaching medical students about the theoretical concept of SDHs and how they impact patients. However, little research has been done on how to teach undergraduate students practical tools that they can use to combat SDHs during their education or practice. The EMPOWER curriculum^17^ is a successful example of training residents to address SDH barriers in a particular patient population; it provided important insights for how to develop and evaluate this workshop. We did not find any existing curriculum evaluations describing a more general training for medical students on how to assess barriers and connect individuals with resources. We consistently received feedback from students at our institution participating in SDH-related lectures and trainings that they wanted more practical information on how they could have an impact, in addition to understanding the concept of SDHs and their effect on care. The case-based workshop described here is intended to train students to actively utilize the material working with community members during service-learning experiences. These acquired tools can help students engage in similar work to address health equity in their future medical careers.

## Methods

### Workshop Development and Content

We designed this workshop as a 90-minute session for medical students to support service-learning activities including helping to connect individuals with community resources and improving access to and relationships with the health care system. We utilized Kern's six-step model^18^ to guide curriculum development. Training content included the following:

• How to assess an individual's barriers to achieving good health and/or accessing health care.
• How to search for appropriate community resources.
• Essential resources in the workshop community addressing needs such as food, housing, legal support, and transportation.
• Basic eligibility criteria and benefits of public health insurance programs.
• Historical basis for mistrust of the health care system in some minority communities, the impact of this mistrust, and what steps medical students and providers could take to develop trusting relationships with their patients.
• Five case studies based on cases encountered during service-learning at Albany Medical College.

We have included sample training slides that can be modified for use at other institutions and in other communities (Appendix A: Training Slides). Where possible, we have also included national resources and/or guidance on how to find appropriate resources in one's local community.

The inclusion of information on medical mistrust as a barrier to health care among minority populations developed out of one author's participation in an intensive student summer internship at a community engagement site in a majority minority population neighborhood in downtown Albany, New York. Conversations surrounding mistrust of medicine arose during several discussions with clients at the organization, who cited both historical incidents of medical mistreatment of people of color and individual negative experiences with care providers in the area. This prompted in-depth research on the topic and, ultimately, the development of training tools to support other students working in similar settings. We created this workshop to support students engaged in service-learning activities related to community resources and accessing the health care system, but it was also intended to provide participants with tools that would be useful during their clinical years and in their future medical practice, no matter where they ended up practicing.

Additional details on workshop implementation can be found in Appendix B: Facilitation Guide.

### Evaluation

We received institutional review board approval (Albany Medical College protocol #4999) to evaluate the workshop using anonymous student pre- and postsurveys completed in Qualtrics (Appendices C: Session Evaluation and D: Evaluation Answer Key). Surveys were designed to measure knowledge, perceived knowledge, and confidence in skills related to the workshop.

The presurvey could be completed any time from 1 week to 1 hour prior to the workshop. It included nine items measuring students' self-evaluation of their confidence and knowledge regarding the material covered in the presentation. We used five Likert-scale response anchors: Strongly Agree, Agree, Neither Agree nor Disagree, Disagree, and Strongly Disagree. The presurvey also included eight questions to measure students' knowledge of the workshop content using multiple-choice and true/false questions.

The postsurvey could be completed any time from immediately after the workshop up to 2 weeks later. It included the same questions as the presurvey as well as asking students if they had gained new knowledge from the training, if they could see themselves using the content during service-learning and/or their future medical career, and if there was anything they would have liked to learn more about.

### Replication

This type of training is, by its nature, specific to the area in which it is being conducted. Insurance eligibility and coverage can differ across states, and the community resources available vary by community. We have included template slides along with notes on what information can be standardized (federal benefits, national resource databases) and what information needs to be tailored (Appendix A). Workshop facilitators can seek help from colleagues in public health, social work, and community outreach to find the information needed to tailor the slides. Additionally, student workers or interns could be recruited to search for community resources and populate the slides, which would provide a more in-depth learning experience.

Specific funding was not received for this training or evaluation, but funds from the Touhey Family Foundation support some of the staff and programmatic work related to the training.

## Results

**Figure 1.**
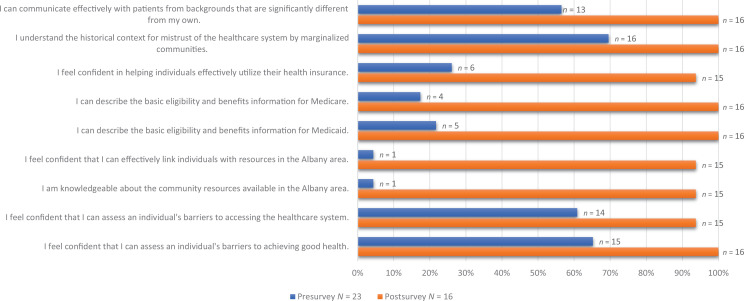
Pre-/postsurvey self-assessment: percent of respondents selecting Agree or Strongly Agree.

Twenty-three students (92%) completed the presurvey, and 16 students (64%) completed the postsurvey. Student responses on the presurvey were scattered, with the majority of students rating their confidence and knowledge in the Agree, Neither Agree nor Disagree, and Disagree categories for all measures (Figure 1). Students rated themselves particularly low in their knowledge and confidence with community resources, with 69% of respondents selecting Disagree or Strongly Disagree for the statement “I am knowledgeable about the community resources available in the Albany area” and 70% of respondents selecting Disagree or Strongly Disagree for the statement “I feel confident that I can effectively link individuals with resources in the Albany area.” On the postsurvey, student responses shifted towards the Agree and Strongly Agree categories in all measures, indicating increased confidence and perceived knowledge. For the questions related to community resources, 94% of postsurvey respondents selected Agree or Strongly Agree for the statements “I am knowledgeable about the community resources available in the Albany area” and “I feel confident that I can effectively link individuals with resources in the Albany area.” One-tailed *t* tests were performed comparing the average pre- and posttest responses for each item on the Likert scale. Each test was statistically significant (*p* < .05).

**Figure 2.**
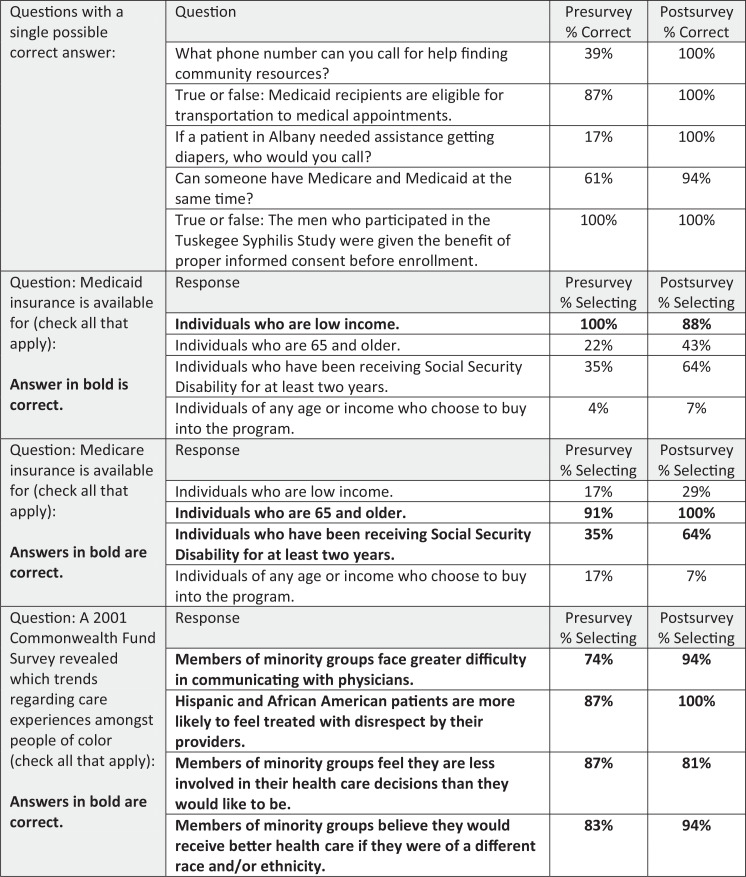
Responses to knowledge-based questions.

As far as the knowledge-based questions, we saw substantial increases in the percentage of students answering correctly, particularly with questions related to specific resources such as diapers (17% correct on the presurvey vs. 100% correct on the postsurvey) and community hotlines (39% correct on the presurvey vs. 100% correct on the postsurvey; Figure 2). With the questions related to mistrust of the health care system, most students answered correctly even on the presurvey, with some increases on the postsurvey (Figure 2). Knowledge questions were cumulatively scored as the number correct out of eight total questions. Using a one-tailed *t* test, posttest scores were significantly higher (*p* < .05) than pretest scores.

All postsurvey respondents indicated that they had learned new information from the training, with most citing the scope of local resources and several mentioning the health insurance information. All postsurvey respondents reported that they expected to use the information in service-learning and in their future medical practice, which is exciting when thinking about the potential long-term impacts of this type of training.

## Discussion

This case-based workshop takes the concepts of SDHs and provides a practical application that medical students can integrate in their future careers. While this training was designed with a certain geographical area in mind, students will be prepared to seek out this information wherever they practice medicine and use these skills to find appropriate partners at other facilities. Involving multiple stakeholders including faculty, students, and community partners enhanced the quality of this training and allowed the content to be applicable in real-time scenarios. Institutions planning to implement the workshop would benefit from having a student and representative from a community service agency review the content and recommend relevant local additions.

The training covers a significant amount of information, including material unfamiliar to students. Features of health insurance coverage and eligibility, particularly for public programs such as Medicare and Medicaid, are important for students to understand. However, details surrounding these topics may be too complex for this type and length of training. On the survey questions regarding Medicaid and Medicare eligibility, some students demonstrated confusion between the two plans on the posttest (Figure 2). This may indicate that the insurance eligibility information was not presented clearly or that the content was too complicated for the type and length of workshop, especially with the number of other materials covered. It might be beneficial to deliver this information differently, address it more directly in the case studies, or reinforce it in another part of the medical curriculum, including in other service-learning experiences. Conversely, on the questions related to the historical basis for mistrust of the health care system, most students selected correct answers in both the pre- and postsurveys. This may indicate that the students taking the training were already familiar with these concepts and that we could increase the depth of the discussion and incorporate new and more challenging content. Students increased their self-ratings of confidence and knowledge in this area from the pre- to the postsurvey on the Likert-scale question, which indicated that even if they entered the training with more knowledge related to mistrust, they still gained something from the discussion. The specific examples included in the training were chosen because they were commonly cited by community partners as having a large influence on their perceptions of the health care system. The slide notes include additional, more recent examples that could be incorporated in the future.

Engagement from students varied, especially during interactive case study discussions. Training sessions scheduled with smaller groups of service-learning students allowed for more focused discussions and fostered a sense of community among collaborating students and faculty. In these focused discussions, students started to express interest in tailored resources and case studies for specific populations such as immigrants, refugees, and the LGBTQ community.

Although the goal of the workshop is to create a foundation of community resources in one's own area, there may not be adequate community resources to meet all SDH needs. Often, certain resources such as those involving safe, affordable housing or medical transportation may have long wait-lists or require certification from a health care provider to be available. Time should be taken to discuss this lack or limited availability of community resources in one's area and to examine how the inaccessibility of these resources might impact the overall health of a future patient, their family, or a community at large.

### Limitations

We delivered the workshop in small service-learning-based groups and conducted this evaluation during the spring semester, when students were less likely to sign up for and become trained in new service-learning activities, which resulted in low overall participant numbers. The response rate for the presurvey was fairly high (92%) but dropped significantly for the postsurvey (64%).

The student participants volunteered to attend the training and were actively involved in service-learning programs related to SDHs and community resource access at the time of the training session. The students' personal interest in this type of program may have increased their level of preexisting knowledge as well as their interest level in the material itself. We did not collect demographic information on the workshop participants. While such information may have been useful, we felt that requiring students to define themselves in this way was not in line with the spirit of the training or related service-learning and could impact the relationship between the service-learning participants and the faculty leading and evaluating the training.

The confidence and knowledge questions in the pre- and postsurveys line up with the topic areas of the educational objectives but in some cases do not go as far as the objectives in measuring actual use of the information from the training. This demonstration of practical skills becomes apparent to some degree in the case study discussion and more fully as the students apply the training content in service-learning and discuss their experiences in reflection.

### Future Directions

Offering a small incentive to students who complete the workshop and both surveys and using different methods for following up with participants might help increase response rates. Diversity of student participants could be improved by including students in service-learning programs not centered around SDHs or embedding the content as part of a mandatory class for all students, such as one focused on ethics or community engagement.

While we have evaluated the impact of the training session, our true intent is for it to be used in a real-life service-learning environment. To that end, a 3-month follow-up evaluation after students have experience in applying the knowledge they acquired during the training is in progress. This is intended to measure the lasting impact of the training, as well as to evaluate whether experience using the information influences student confidence, knowledge, or perceptions of the training.

For the future, this project could be expanded to other students or trainees, including physician assistants, nurse practitioners, and pharmacists, in order to create a greater consortium of well-informed medical providers. In addition to this training evaluation, we are conducting evaluations of community members and patients whom students have worked with during service-learning to document that the students who participated in the training are able to meet the needs of the patients and families they are assisting.

Overall, the training yielded very positive outcomes regarding the knowledge gained by students as well as the expectation of carrying the information learned with them to their future medical practices. SDHs and other barriers to health such as mistrust in the health care system remain vital topics in undergraduate medical education. By familiarizing medical students with these concepts, we give future health care providers the knowledge to actively work with future patients to assess and help remove these barriers. We will continue to build on this curriculum given the strong association between SDHs and the health outcomes of patients.

## Appendices


Training Slides.pptxFacilitation Guide.docxSession Evaluation.docxEvaluation Answer Key.docx
*All appendices are peer reviewed as integral parts of the Original Publication.*
