# Supplementary material for: A Case-Based Workshop Training Medical Students in Assessing Social Determinants of Health Needs and Connecting With Community Resources
Source: MedEdPORTAL. 2022 Mar 21;18:11232. doi: 10.15766/mep_2374-8265.11232 (PMC8934752; doi:10.15766/mep_2374-8265.11232)
Supplement: Supplementary file 1 — Training Slides.pptxFacilitation Guide.docxSession Evaluation.docxEvaluation Answer Key.docx [file mep_2374-8265.11232-s001.zip › B. Facilitation Guide.docx]

Implementation & Case Study Facilitation Guide

**Workshop Implementation:**

Background:

At Albany Medical College, all students are required to participate in service-learning prior to graduation, which goes beyond the LCME (Liaison Committee on Medical Education) requirement that service-learning and community service opportunities be offered to students. Albany Medical College students choose which service-learning activities to participate in from approximately 30 options, and students do the bulk of their service learning during the first and second years. Each program has its own training requirements.

We recruited students from service-learning programs that involve working one-on-one or in small groups to support community members through activities like accessing public benefits, securing, and utilizing health insurance, finding healthcare providers, and addressing the social determinants of health. These service-learning programs include activities working with formerly incarcerated individuals, people experiencing homelessness, immigrants, refugees, asylum-seekers, adolescent mothers, and other underserved groups. We implemented the workshops in small groups of 6-11 students all working in the same service-learning program, which allowed for more targeted questions and resource discussions, as well as more detailed interpretation of the case studies.

Implementation details:

Approximately the first hour of the training consists of PowerPoint slides reviewing the content, with time for students to ask questions and engage with the materials. With three presenters each slide was covered by the individual most familiar with the content, but one facilitator could lead the entire training if necessary. The final 30 minutes is dedicated to discussions of five case studies. Each study is shown on the screen along with a series of questions, and students discuss how they would approach working with the client based on the information covered during the training. Facilitators follow up by responding to student comments and describing how the issue was resolved during the actual case (see below). With groups of up to 11 students we found that discussing the case studies as a group worked well, but for groups larger than 12 we would recommend breaking up into 2-3 subgroups (either virtually or in person using breakout rooms) to discuss and report back.

The workshops described in this paper were co-led by community outreach faculty, staff, and a medical student with experience in service learning. The makeup and number of workshop facilitators can vary, but it should be led by individuals who have experience with community engagement and some basic knowledge of health insurance policy and local community organizations. It was originally designed as an in-person workshop, but the sessions evaluated and discussed in this paper were done virtually via WebEx due to the COVID-19 pandemic.

**Case Study Facilitation:**

After presenting the case studies to the students by showing the slide and reading aloud, allow time for students to produce the possible next steps. If there is a lack of conversation use the prompts below the case study to encourage students to think through the possibilities.

**Case #1- Mr. Jones**

Mr. Jones wants to set up an appointment with a primary care physician. He has never had one before and uses the Emergency Room for most of his care. He is not sure what type of health insurance he has, but thinks he has some.

Based on what you know about this client, what would you do next?

What barriers do you think he might face? What assets might he have?

What community resources or types of resources would be appropriate?

*Facilitator notes:*

- Figure out what type of insurance the client has. Do this by calling your local state or federal health insurance marketplace or Department of Social Services depending on where the client originally signed up for his insurance. Another option would be to three-way call the emergency room the client went to in the past and see if they can look up his information and look at his insurance. If the client states that they have insurance through a job (either their own or a spouse/parent’s) call the human resources department for that job.
- Once you have figured out what insurance the client has you can use the “find-a-doc” feature on the insurance website to find an appropriate doctor. First, check if the client has any preferences of where the provider is affiliated (some clients have had bad experiences with certain facilities and do not want to go back) or any issues with transportation, so you can find a facility that is close.
- Make a list of multiple providers that would work for what the clients’ wants/needs and call each office to double check they take the client’s insurance (sometimes the website is wrong), they are taking new patients, if they have a long wait, and what their cancelation policy is (this is helpful to know for the future).
- Talk with your client to see which provider they would like the most. If the client would like support making the appointment, three-way call the providers office back and set up an appointment with the client on the phone.
- Talk with your client about a plan for how to get to the appointment. If they have Medicaid, they will be eligible for transportation support to the appointment (public transportation or a taxi ride, depending on their state and qualifying conditions). If they do not have Medicaid, you can look up an easily accessible bus route. After the appointment is set up it might be useful to talk with the client and write a list of all the things that they would like to discuss during their appointment. For example, they may have specific symptoms they would like to discuss or questions about medications. Another example of this would be if they have transportation issues and need a ride through Medicaid transportation services, they may need their doctor to state they are unable to take public transportation because of a physical/mental/social complication to allow for them to take a free taxi instead.

**Case #2 – Ms. Smith**

Ms. Smith moved to Albany from South Carolina a few years ago. She is trying to collect her medical records from all her past providers so that she has a complete copy herself but is having trouble getting the various offices to send her records. She thinks she may have been enrolled in a governmental study and former President Obama is preventing her from accessing her records.

Based on what you know about this client, what would you do next?

What barriers do you think she may face? What about assets?

What community resources or types of resources would be appropriate?

*Facilitator notes:*

- Call all the previous providers with the client on a three-way call and ask to send all her current medical records to her so she can continue her care in another state.
- In this case, the client had very difficult providers who refused to send her medical records. At this point we had to utilize a lawyer, who volunteers with us, to write a letter stating that these providers must send this client all her medical records. The providers sent the medical records after receiving this letter, but also sent pieces of another individuals records as well. In that case, medical students assisted the client in filing a HIPAA complaint against that facility through the department of Health and Human Services <https://www.hhs.gov/hipaa/filing-a-complaint/index.html> (URL is optional).
  - If you do not have a lawyer at your disposal, you can try connecting with a local law school or see if there are any legal aid organizations in your area.
- This client had also mentioned she was in a governmental study, and it turns out that she was, but that had nothing to do with her medical records being withheld.
  - Avoid discrediting her beliefs that she was in a governmental study and that President Obama is the one withholding her medical records. Use this as an opportunity to discuss mistrust in the healthcare system.
- Once the client has the medical records, you can ask if they would like help with the continuation of her care by helping her look for and set up future doctors’ appointments.
  - Keep in mind provider preferences, transportation, and assisting the client in prioritizing their medical needs.

**Case #3- Mr. Bass**

Mr. Bass has Medicare/Medicaid. He needs to reschedule a surgery that he missed because he did not know he needed a preoperative physical. He also needs assistance with transportation to his surgery and all pre-op and post-op appointments.

Based on what you know about this client, what would you do next?

What barriers do you think he may face? What assets might he have?

What community resources or types of resources would be appropriate?

*Facilitator notes:*

- Call back the providers and reschedule the surgery appointment as well as the preoperative physical. You should also check and make sure the client does not need to schedule any other pre- or post-appointments regarding their surgery.
  - Be prepared to advocate on the clients behave to reschedule the missed appointments. Some facilities have very strict cancellation/no show policies.
- At this point it might be helpful to compose a document for the client that has all their upcoming appointments on a calendar (paper or electronic, whichever the client prefers), so they are able to easily keep track of their appointments and what they might need to bring with them.
- Check to see if the client has transportation. If they do not and have Medicaid, they are able to utilize the Medicaid transportation services. You will need to call the provider and ask them to document that they are unable to take public transportation due to a physical/mental/ social complication for a free taxi and schedule the ride a couple of days before each appointment. If they are not eligible for a free taxi ride, they will get free bus passes mailed to them.

**Case #4- Mrs. Apple**

Mrs. Apple’s daughter was told by one of her teachers that she has dyslexia, but the teacher said that her insurance (CDPHP Medicaid) will not pay for testing. She needs an official diagnosis to get any services or special accommodations at school.

- - Based on what you know about this client, what would you do next?
  - What barriers do you think she may face? What assets might she have?
  - What community resources or types of resources would be appropriate?

*Facilitator notes:*

- Call the clients insurance company and make sure that they do not cover testing services for dyslexia.
- In this case the insurance company did cover those testing services as long as they were performed in a primary care setting, but the daughter’s teacher had told the client misinformation.
  - If the insurance company did not cover the testing services, you could have reached out to the daughter’s school to see if they knew of any local resources or looked in a national association who sometimes have grant money to help with these kinds of things.
- You can ask the client if they would like help setting up the testing appointment or if they need assistance with transportation.

**Case #5- Ms. Smith**

Ms. Smith is a 27-year-old woman who works at Albany Med as an ER nurse. She completes the social determinants screening form while at a 12-month pediatric appointment for her daughter. She checks off that she has sometimes been unable to get medications for herself or her family members due to cost. She also indicates that she needs help getting diapers.

Based on what you know about this client, what would you do next?

What barriers might she face? What about assets?

What community resources or types of resources would be appropriate?

*Facilitator notes:*

- Call the client and make sure that diapers and medication costs were the only thing that they needed assistance with.
- For diapers, you can research who the national diaper bank provider in the area is or consider local pantries who might also have diapers.
- For medication costs we utilize a local pharmacy college that has a student run program that assists with medication costs by finding cheaper alternatives, etc. Consider any local pharmacy colleges in your area or call the clients insurance company and/or prescribing physician to see if there are any generic brands of the medication that the client could get at a lower cost.
