# Supplementary material for: A Case-Based Workshop Training Medical Students in Assessing Social Determinants of Health Needs and Connecting With Community Resources
Source: MedEdPORTAL. 2022 Mar 21;18:11232. doi: 10.15766/mep_2374-8265.11232 (PMC8934752; doi:10.15766/mep_2374-8265.11232)
Supplement: Supplementary file 1 — Training Slides.pptxFacilitation Guide.docxSession Evaluation.docxEvaluation Answer Key.docx [file mep_2374-8265.11232-s001.zip › A. Training Slides.pptx]

## Slide 1
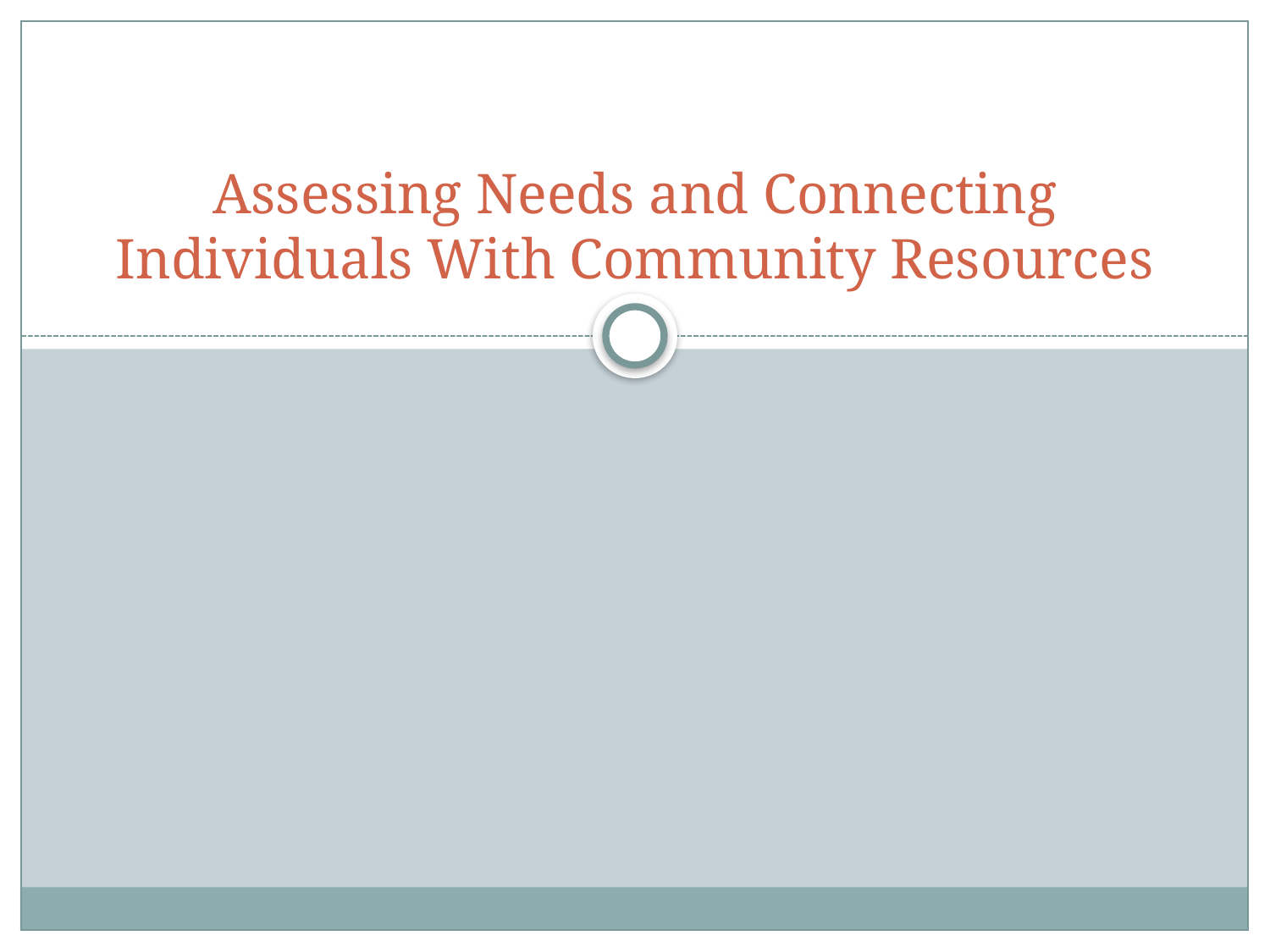

# Assessing Needs and Connecting Individuals With Community Resources

## Slide 2
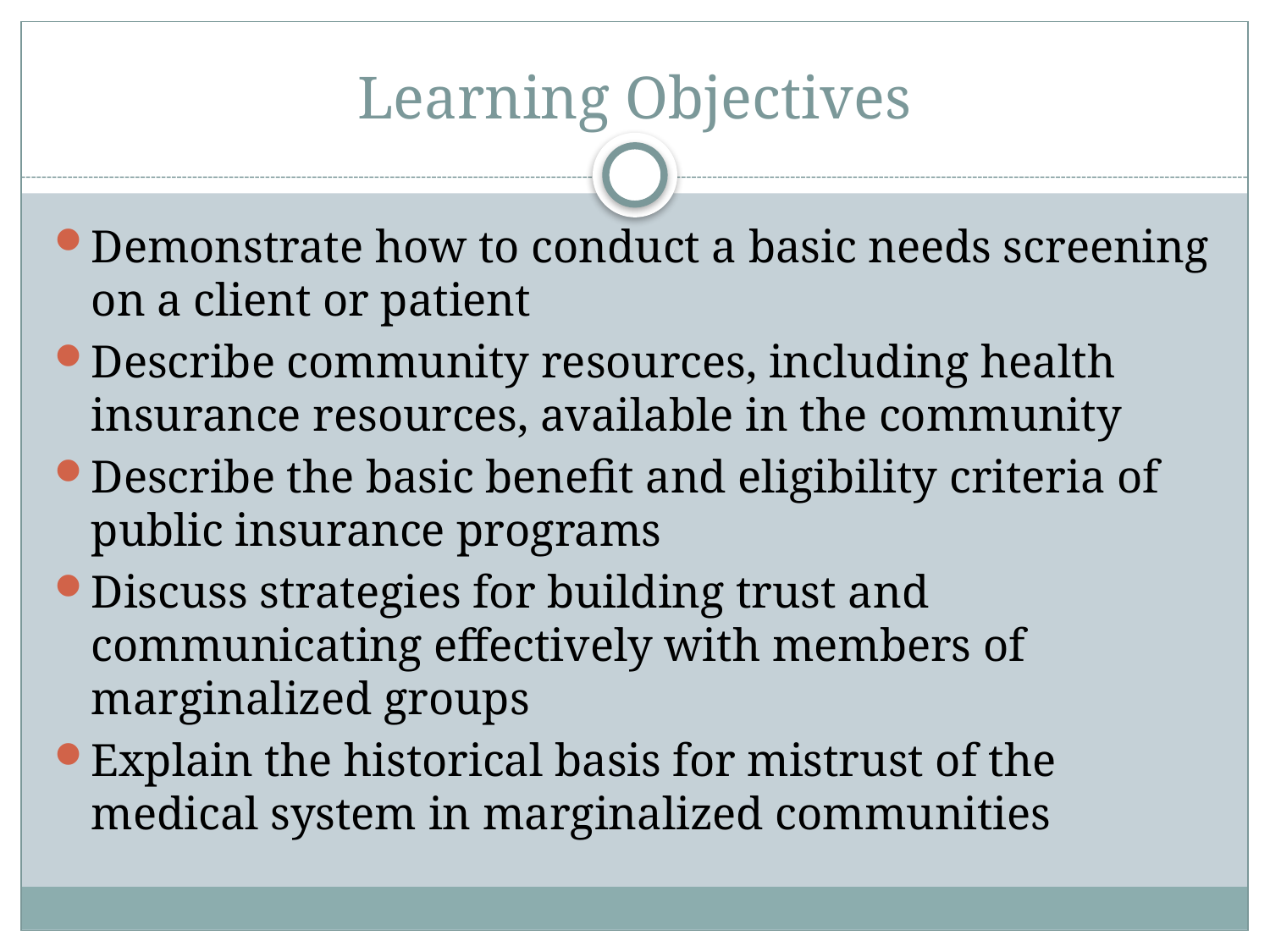

# Learning Objectives
Demonstrate how to conduct a basic needs screening on a client or patient
Describe community resources, including health insurance resources, available in the community
Describe the basic benefit and eligibility criteria of public insurance programs
Discuss strategies for building trust and communicating effectively with members of marginalized groups
Explain the historical basis for mistrust of the medical system in marginalized communities

## Slide 3
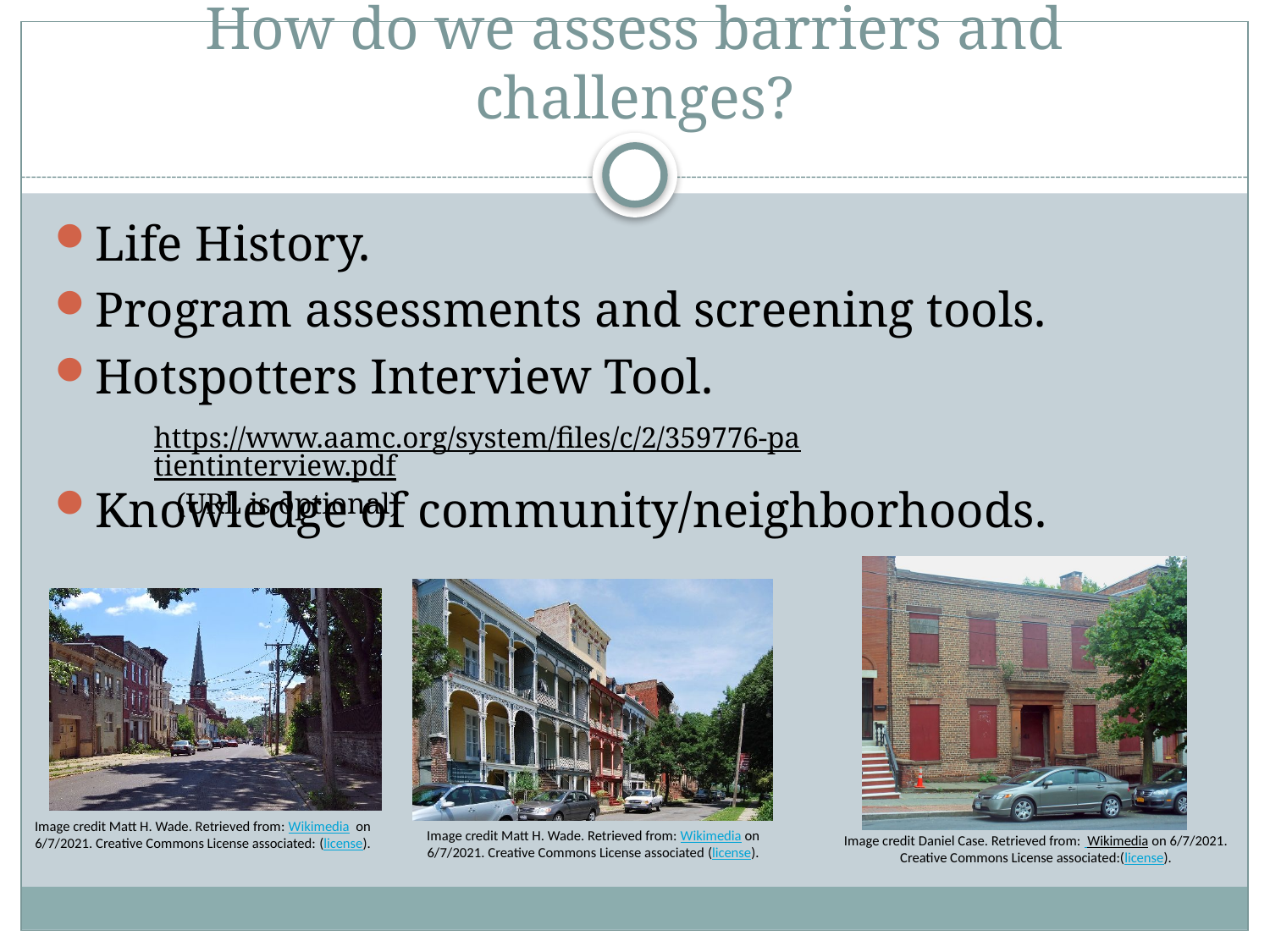

# How do we assess barriers and challenges?
Life History.
Program assessments and screening tools.
Hotspotters Interview Tool.
Knowledge of community/neighborhoods.
https://www.aamc.org/system/files/c/2/359776-patientinterview.pdf   (URL is optional)
Image credit Matt H. Wade. Retrieved from: Wikimedia  on 6/7/2021. Creative Commons License associated: (license).
Image credit Matt H. Wade. Retrieved from: Wikimedia on 6/7/2021. Creative Commons License associated (license).
Image credit Daniel Case. Retrieved from:  Wikimedia on 6/7/2021. Creative Commons License associated:(license).

## Slide 4
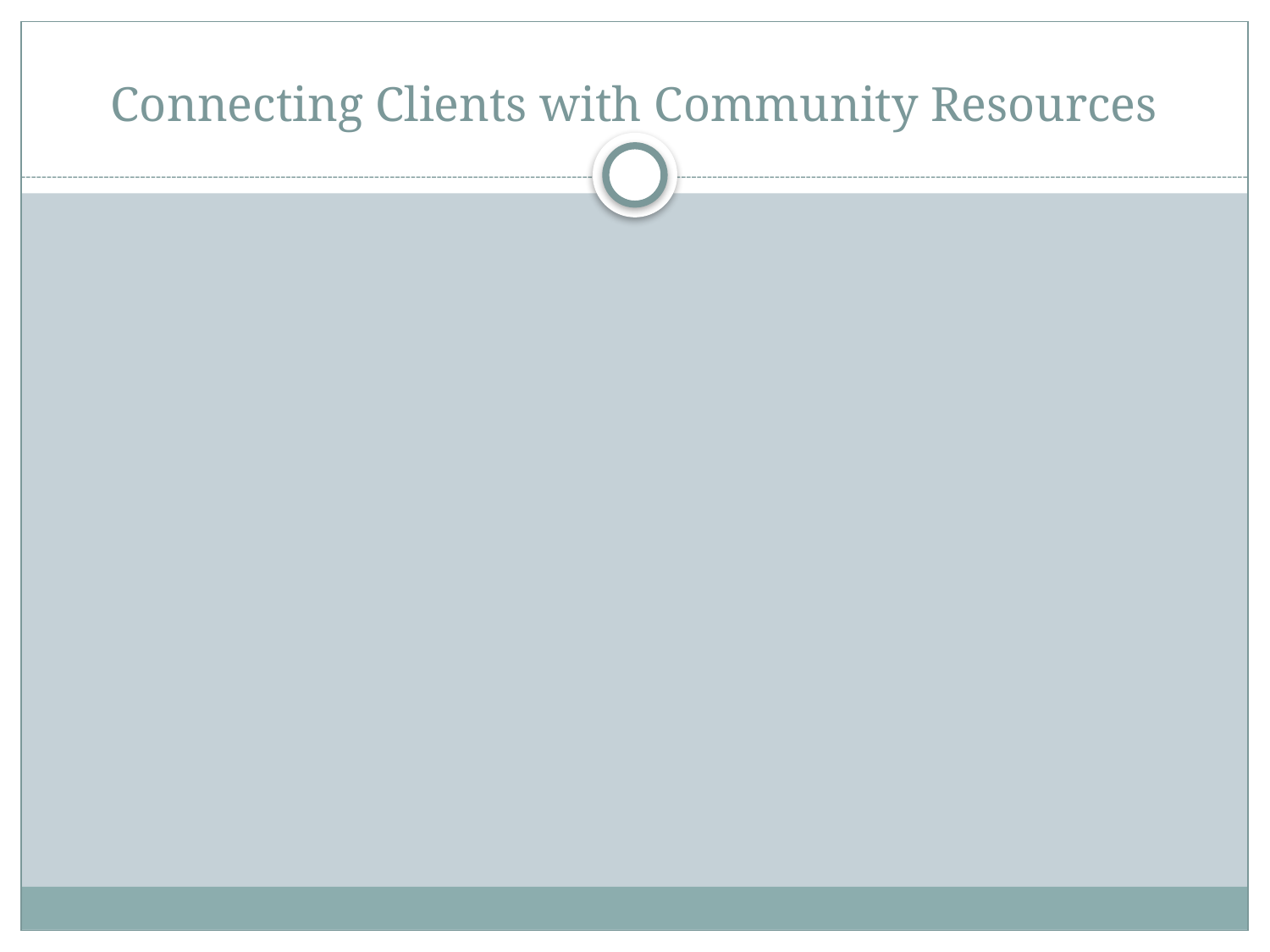

# Connecting Clients with Community Resources

## Slide 5
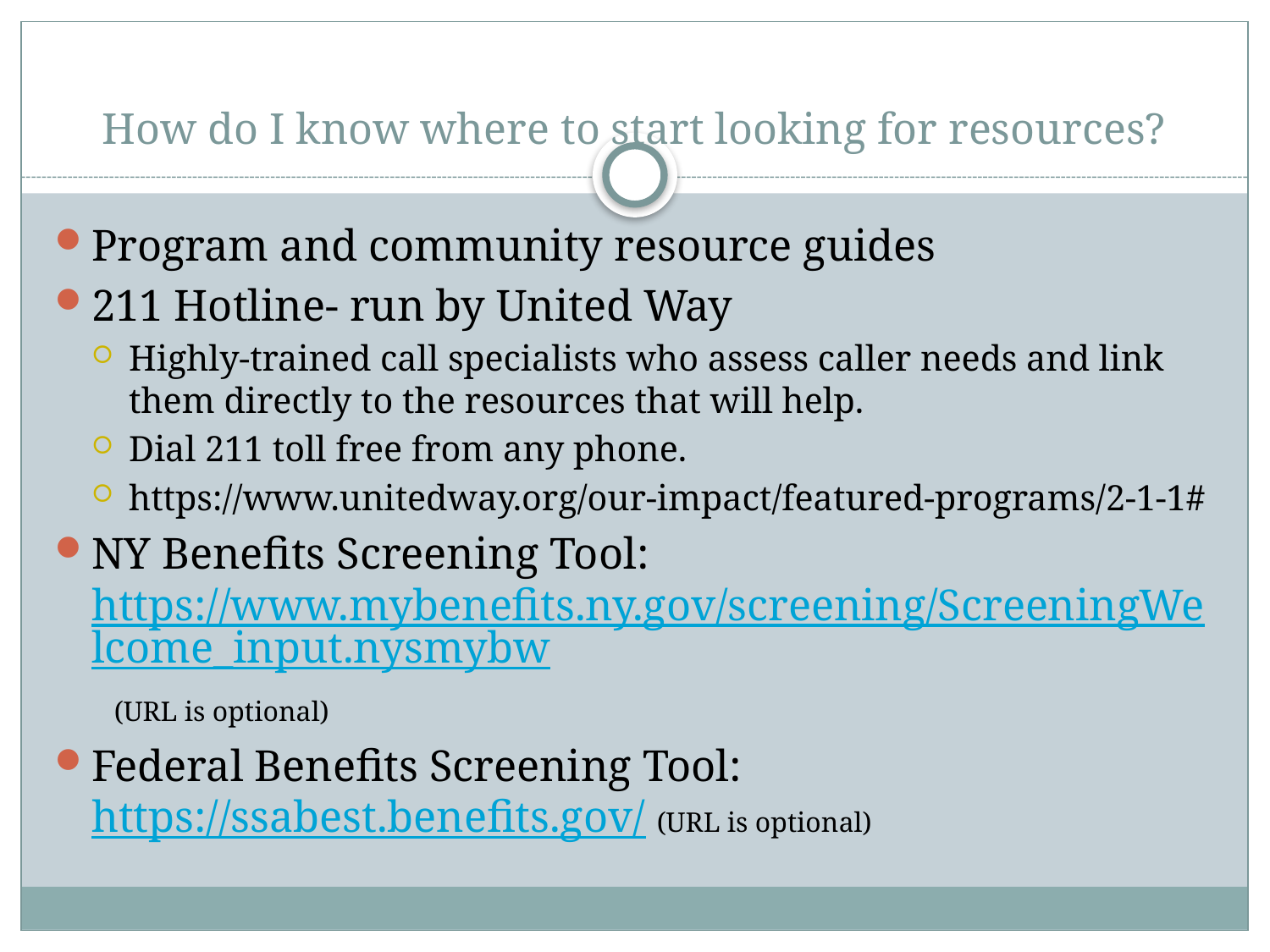

# How do I know where to start looking for resources?
Program and community resource guides
211 Hotline- run by United Way
Highly-trained call specialists who assess caller needs and link them directly to the resources that will help.
Dial 211 toll free from any phone.
https://www.unitedway.org/our-impact/featured-programs/2-1-1#
NY Benefits Screening Tool:   https://www.mybenefits.ny.gov/screening/ScreeningWelcome_input.nysmybw  (URL is optional)
Federal Benefits Screening Tool:  https://ssabest.benefits.gov/ (URL is optional)

## Slide 6
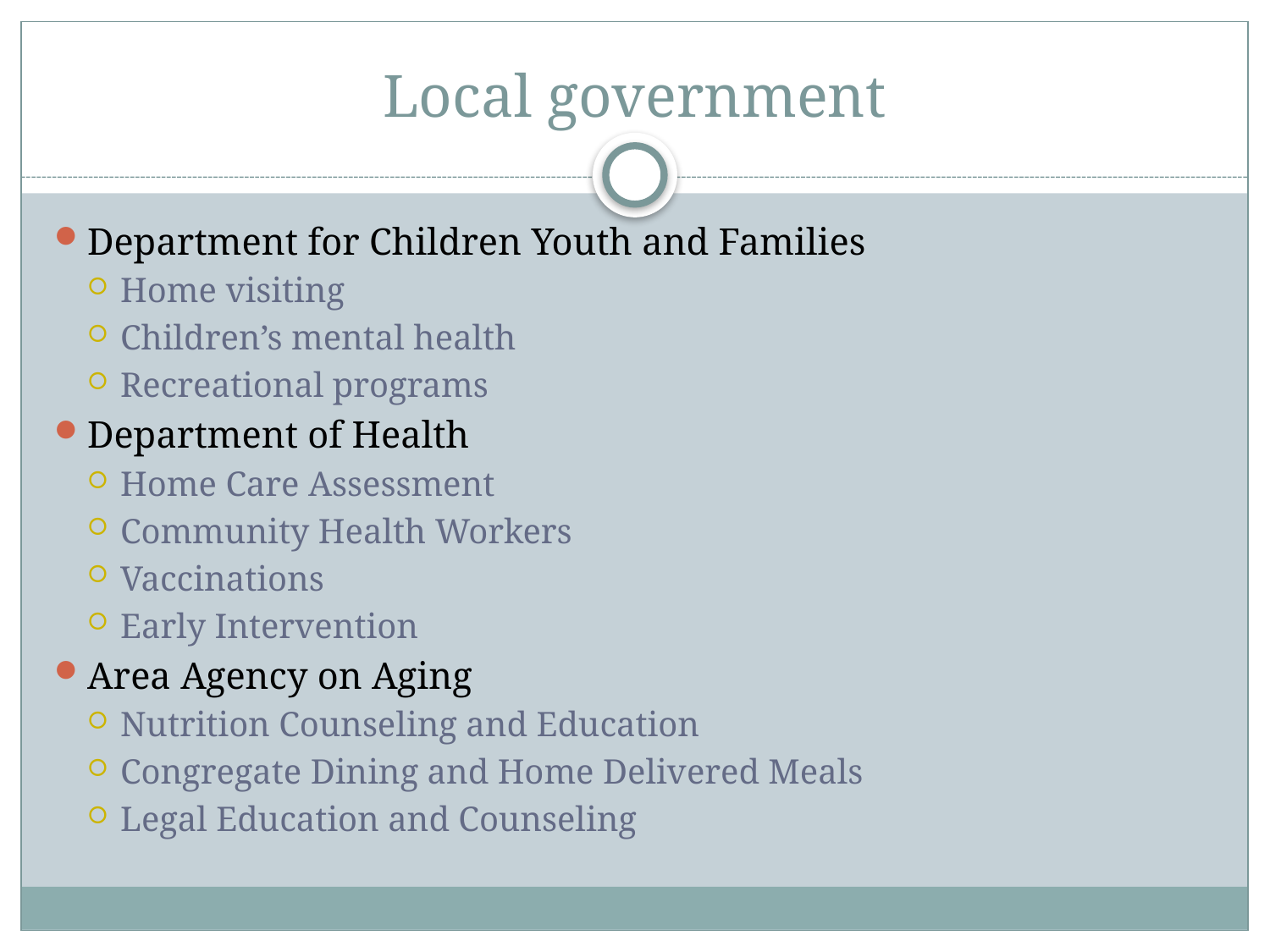

# Local government
Department for Children Youth and Families
Home visiting
Children’s mental health
Recreational programs
Department of Health
Home Care Assessment
Community Health Workers
Vaccinations
Early Intervention
Area Agency on Aging
Nutrition Counseling and Education
Congregate Dining and Home Delivered Meals
Legal Education and Counseling

## Slide 7
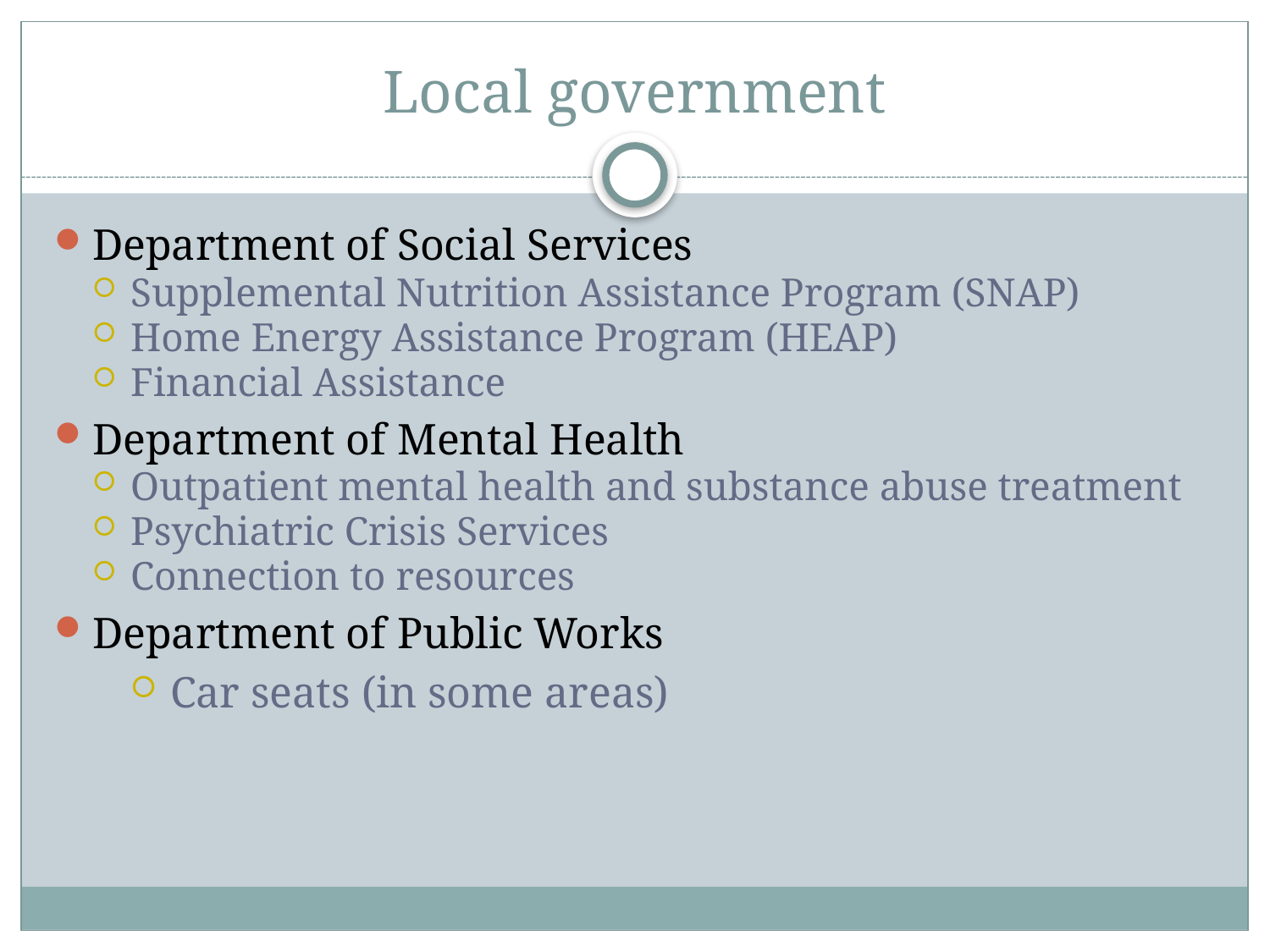

# Local government
Department of Social Services
Supplemental Nutrition Assistance Program (SNAP)
Home Energy Assistance Program (HEAP)
Financial Assistance
Department of Mental Health
Outpatient mental health and substance abuse treatment
Psychiatric Crisis Services
Connection to resources
Department of Public Works
Car seats (in some areas)

## Slide 8
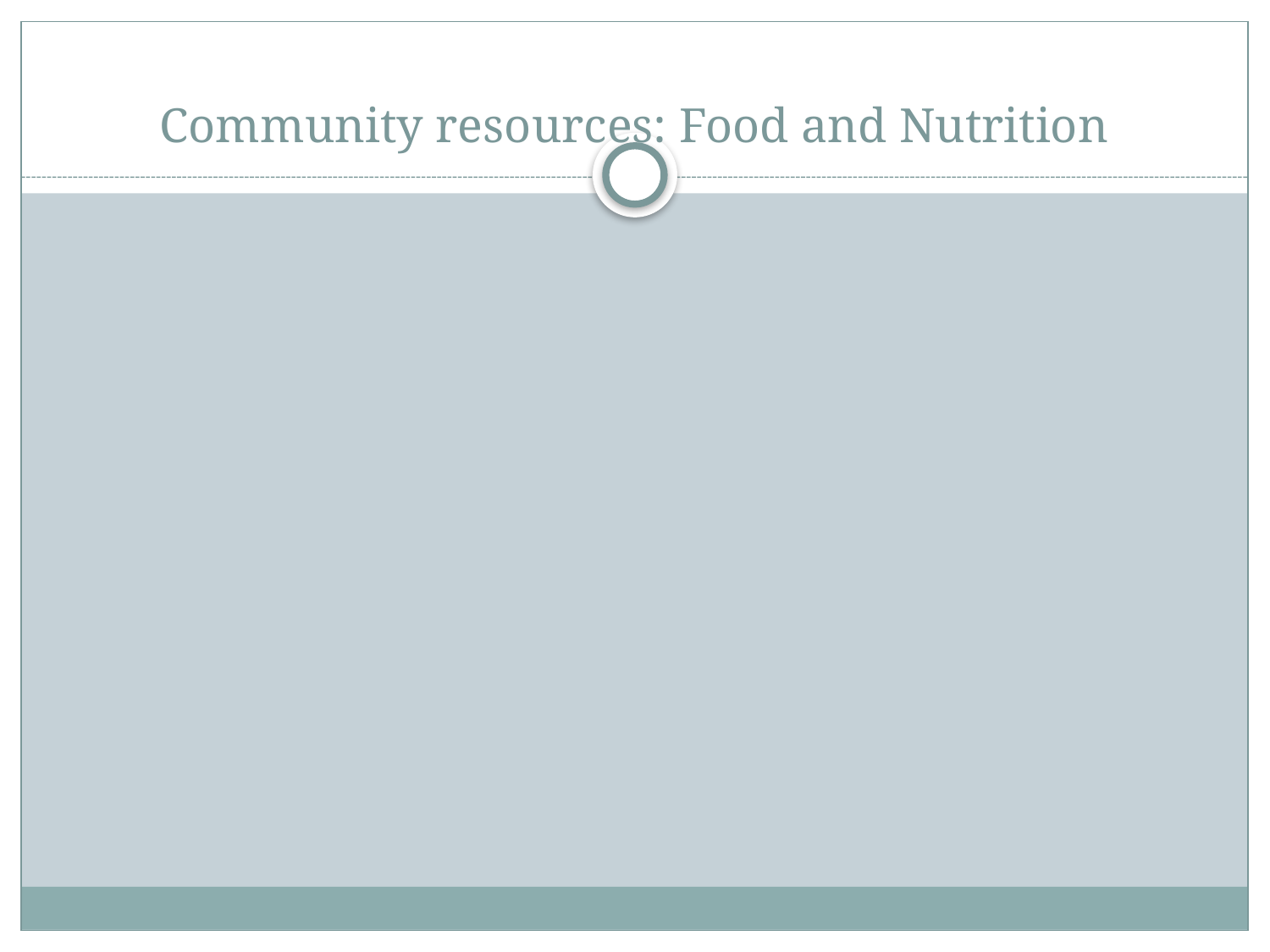

# Community resources: Food and Nutrition

## Slide 9
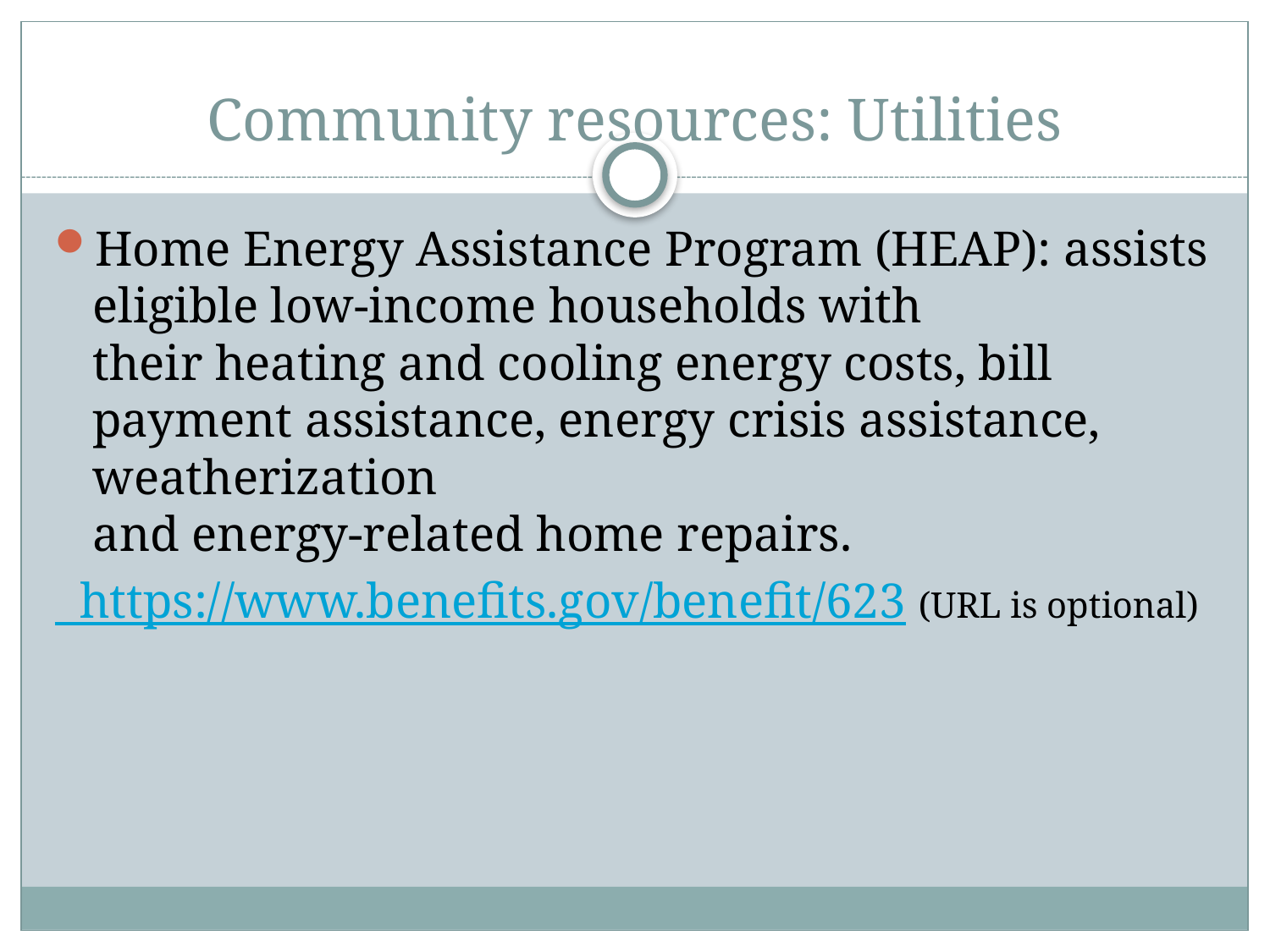

# Community resources: Utilities
Home Energy Assistance Program (HEAP): assists eligible low-income households with
their heating and cooling energy costs, bill payment assistance, energy crisis assistance, weatherization
and energy-related home repairs.
  https://www.benefits.gov/benefit/623 (URL is optional)

## Slide 10
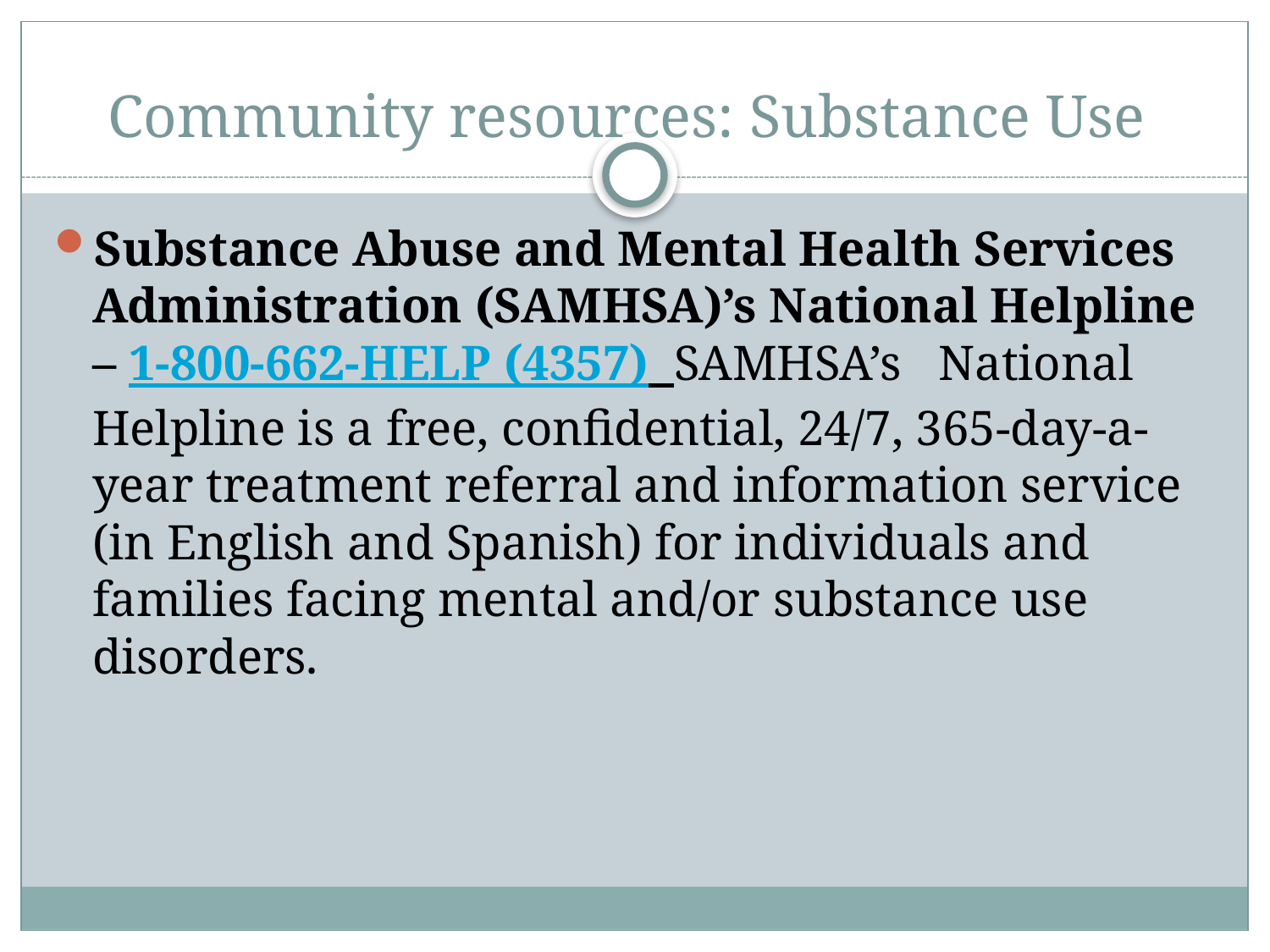

# Community resources: Substance Use
Substance Abuse and Mental Health Services Administration (SAMHSA)’s National Helpline – 1-800-662-HELP (4357) SAMHSA’s National Helpline is a free, confidential, 24/7, 365-day-a-year treatment referral and information service (in English and Spanish) for individuals and families facing mental and/or substance use disorders.

## Slide 11
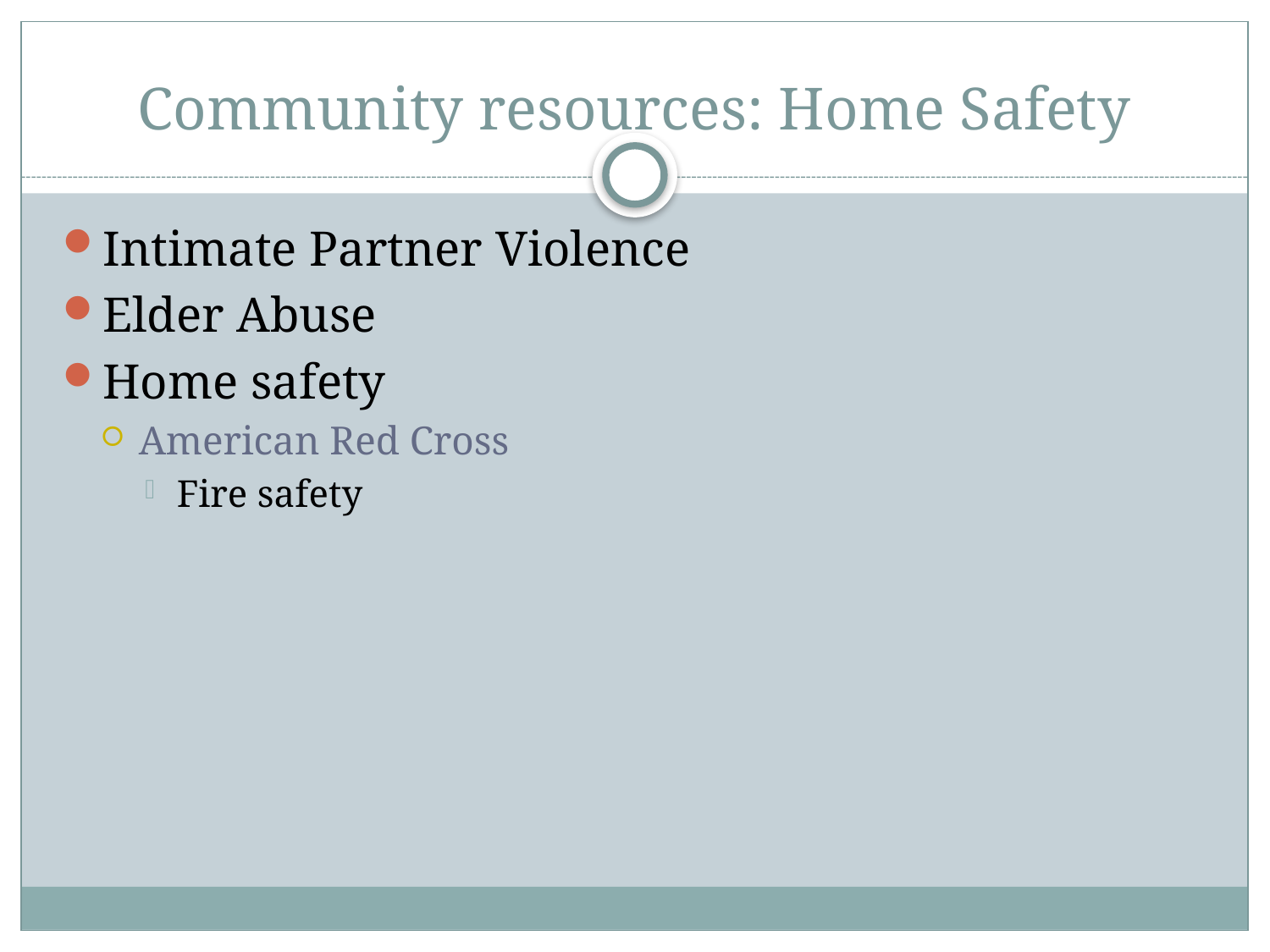

# Community resources: Home Safety
Intimate Partner Violence
Elder Abuse
Home safety
American Red Cross
Fire safety

## Slide 12
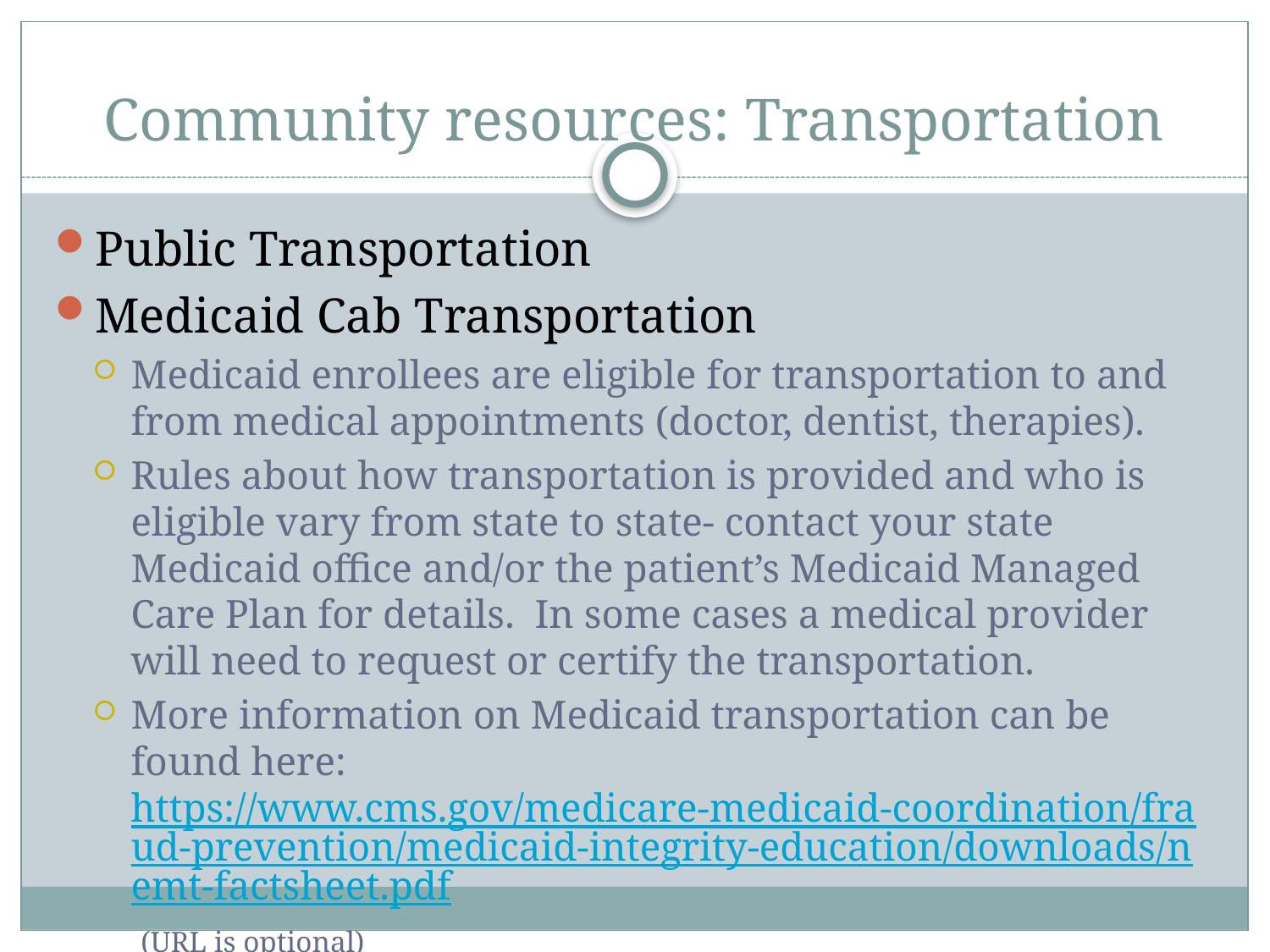

# Community resources: Transportation
Public Transportation
Medicaid Cab Transportation
Medicaid enrollees are eligible for transportation to and from medical appointments (doctor, dentist, therapies).
Rules about how transportation is provided and who is eligible vary from state to state- contact your state Medicaid office and/or the patient’s Medicaid Managed Care Plan for details.  In some cases a medical provider will need to request or certify the transportation.
More information on Medicaid transportation can be found here: https://www.cms.gov/medicare-medicaid-coordination/fraud-prevention/medicaid-integrity-education/downloads/nemt-factsheet.pdf (URL is optional)

## Slide 13
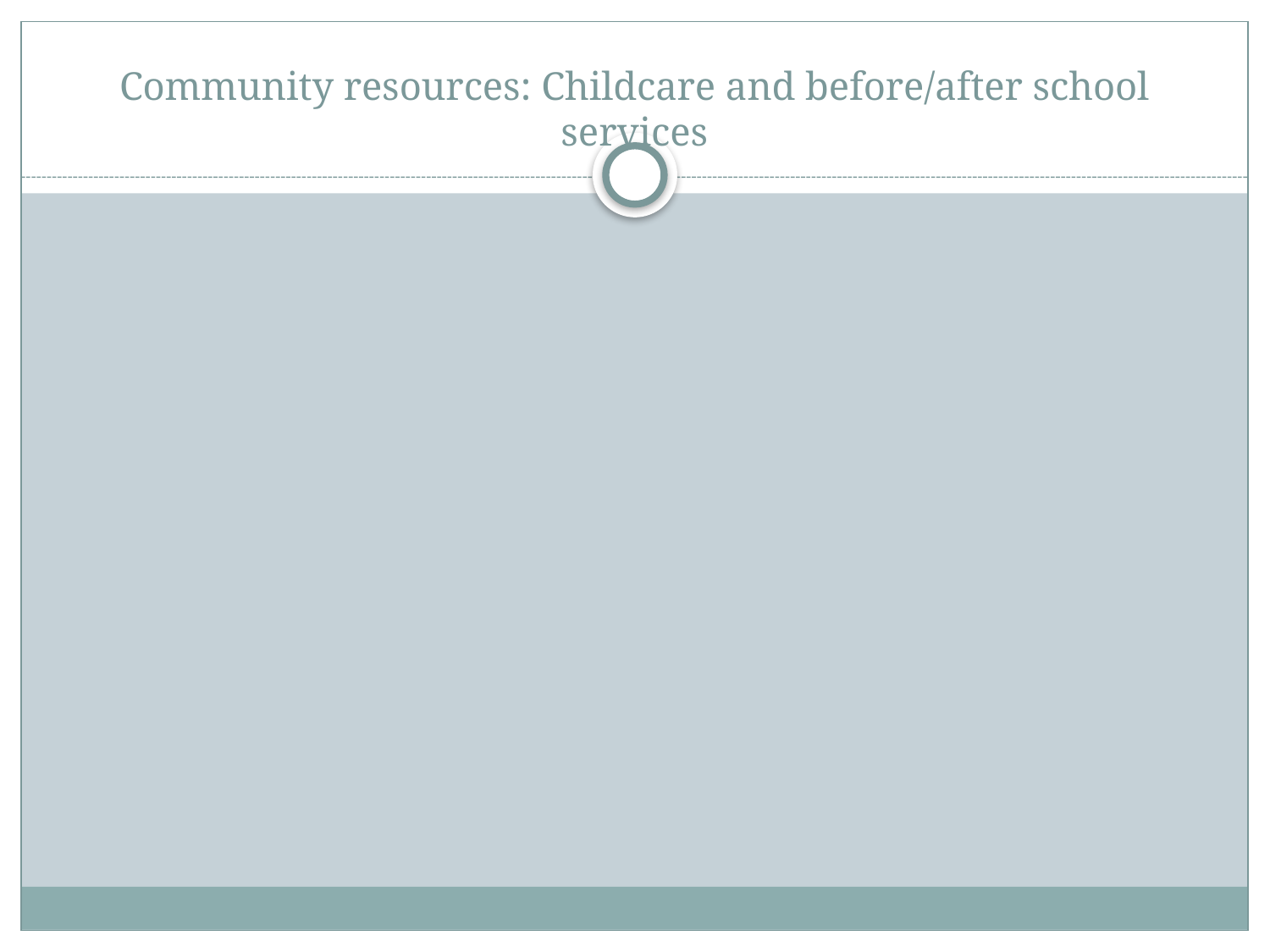

# Community resources: Childcare and before/after school services

## Slide 14
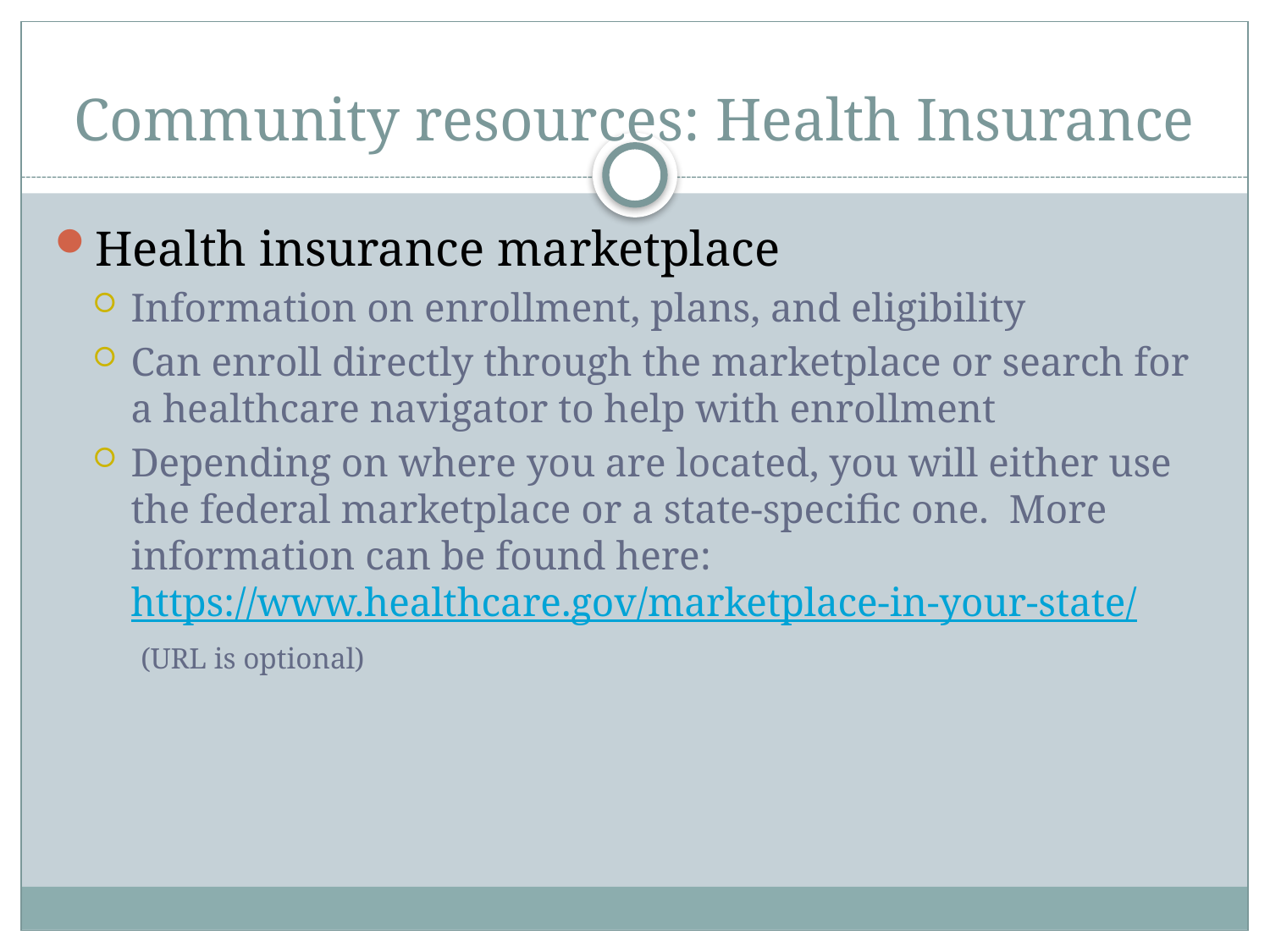

# Community resources: Health Insurance
Health insurance marketplace
Information on enrollment, plans, and eligibility
Can enroll directly through the marketplace or search for a healthcare navigator to help with enrollment
Depending on where you are located, you will either use the federal marketplace or a state-specific one.  More information can be found here: https://www.healthcare.gov/marketplace-in-your-state/ (URL is optional)

## Slide 15
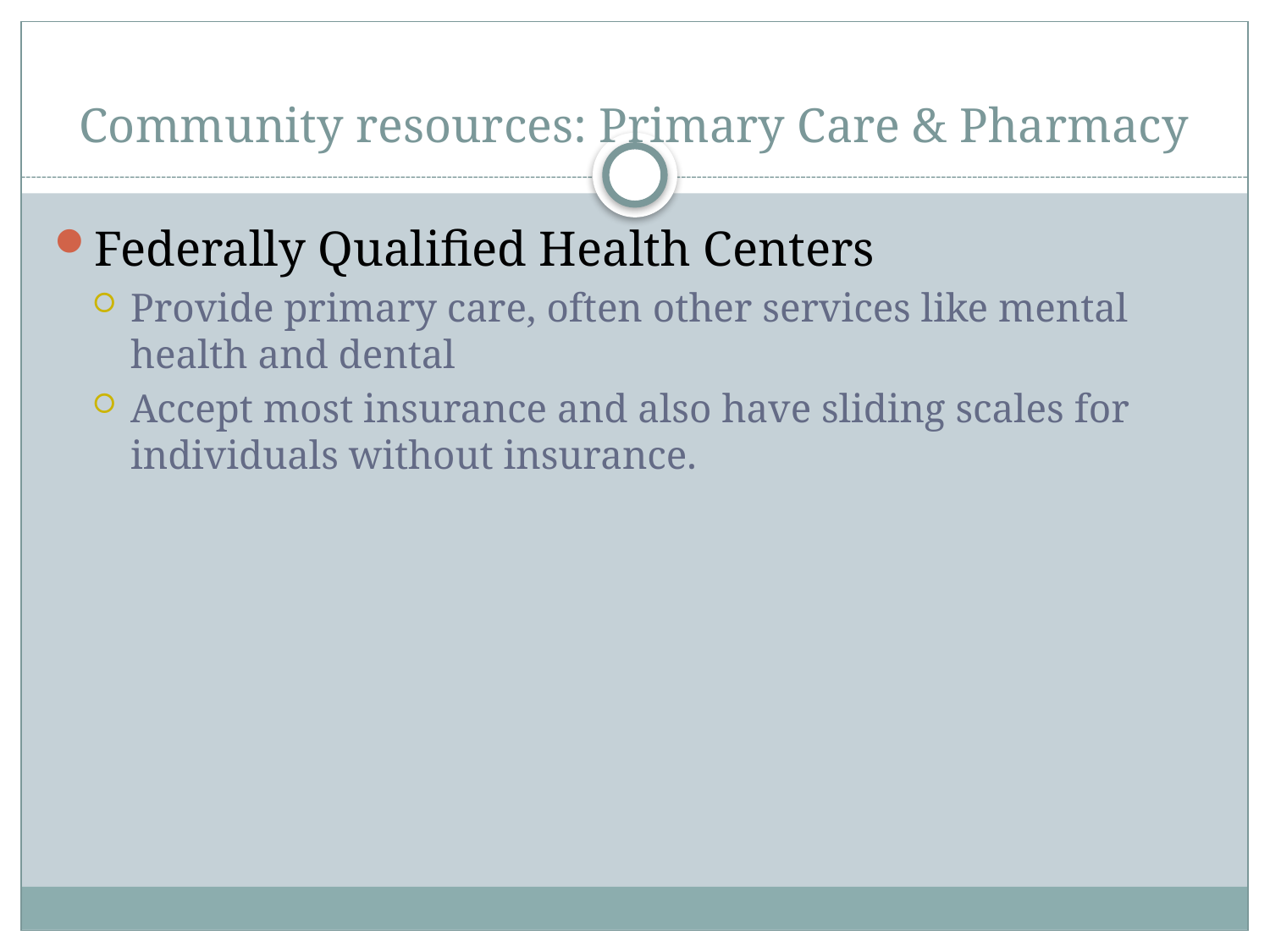

# Community resources: Primary Care & Pharmacy
Federally Qualified Health Centers
Provide primary care, often other services like mental health and dental
Accept most insurance and also have sliding scales for individuals without insurance.

## Slide 16
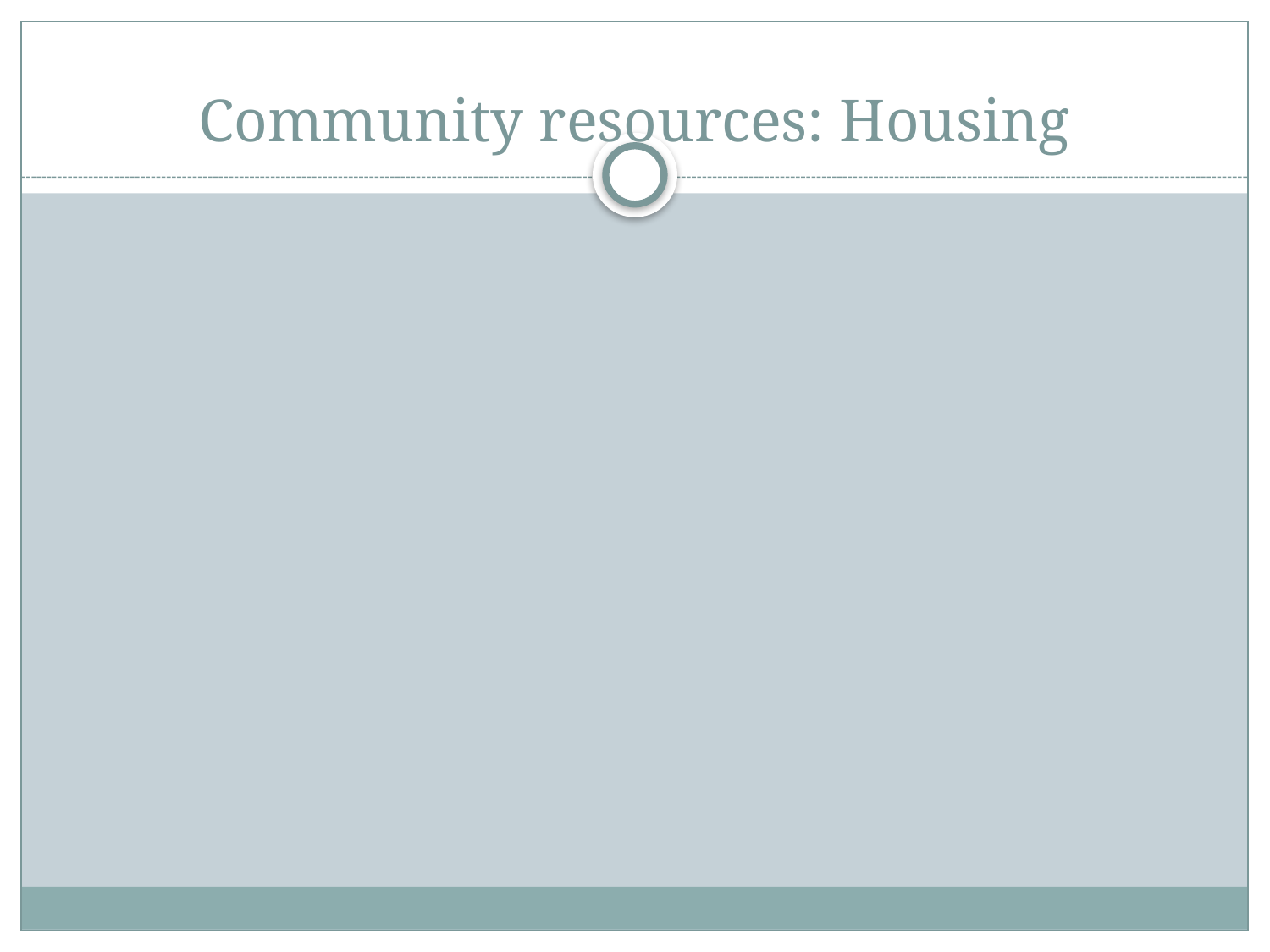

# Community resources: Housing

## Slide 17
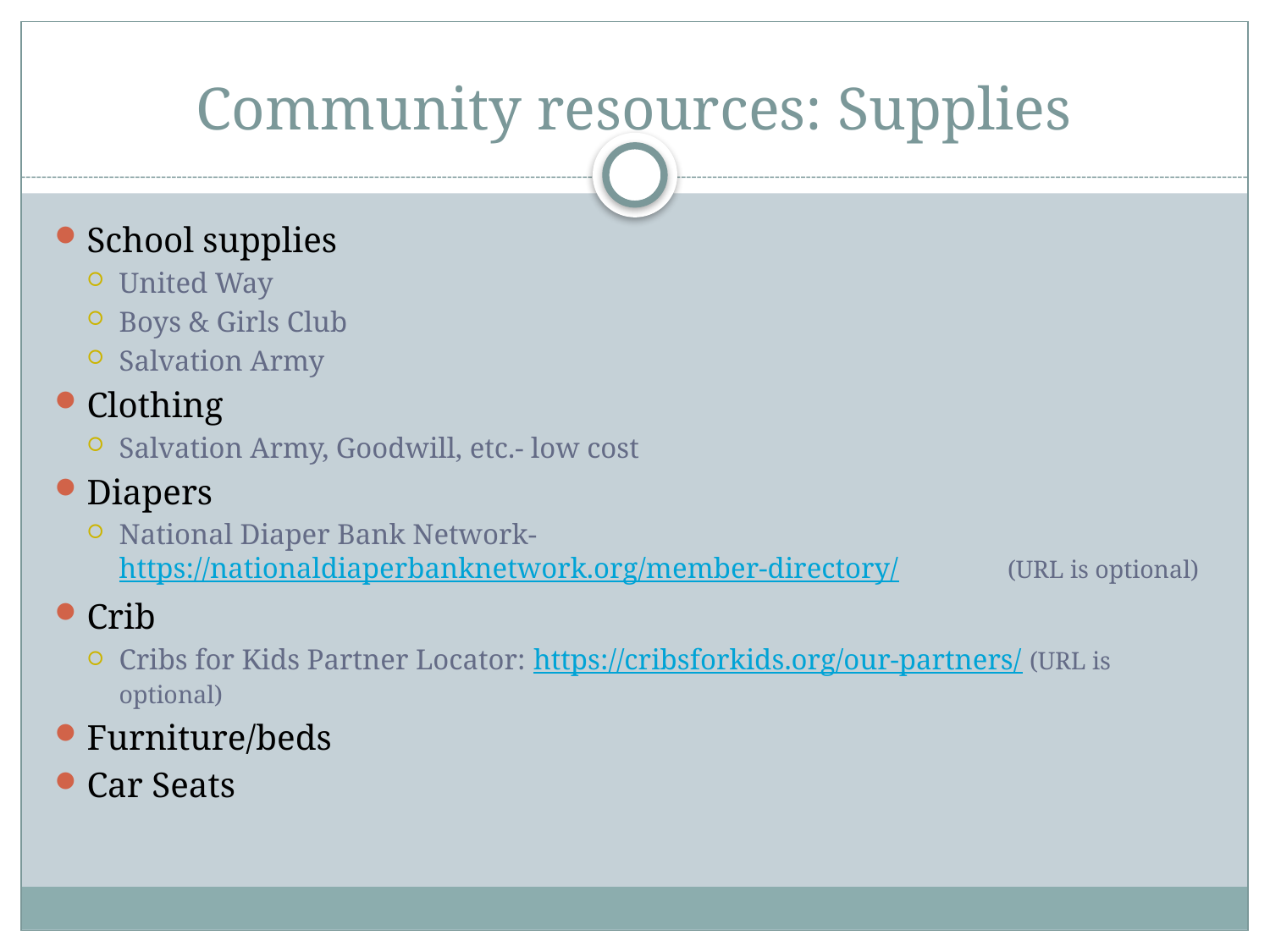

# Community resources: Supplies
School supplies
United Way
Boys & Girls Club
Salvation Army
Clothing
Salvation Army, Goodwill, etc.- low cost
Diapers
National Diaper Bank Network- https://nationaldiaperbanknetwork.org/member-directory/               (URL is optional)
Crib
Cribs for Kids Partner Locator: https://cribsforkids.org/our-partners/ (URL is optional)
Furniture/beds
Car Seats

## Slide 18
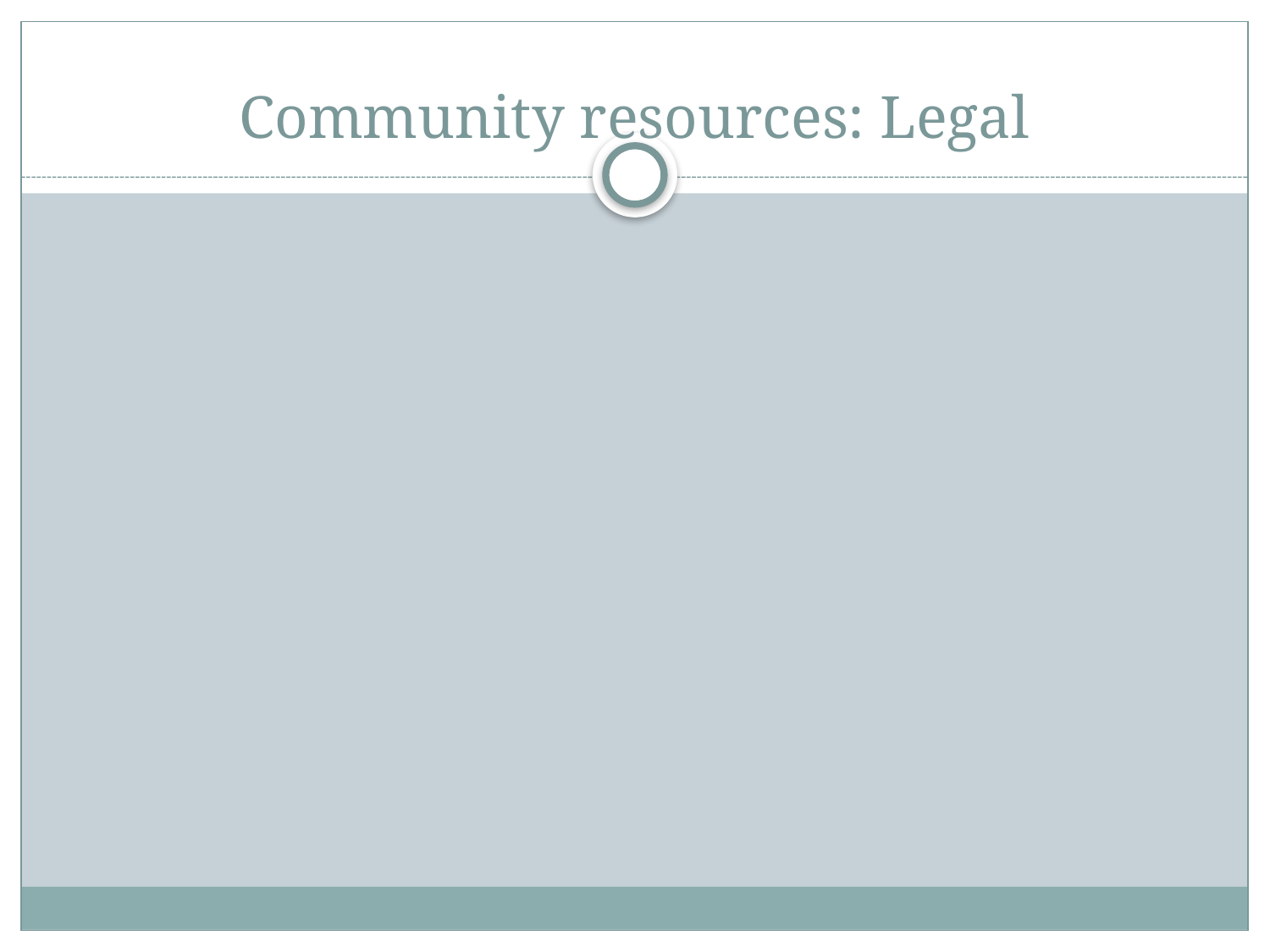

# Community resources: Legal

## Slide 19
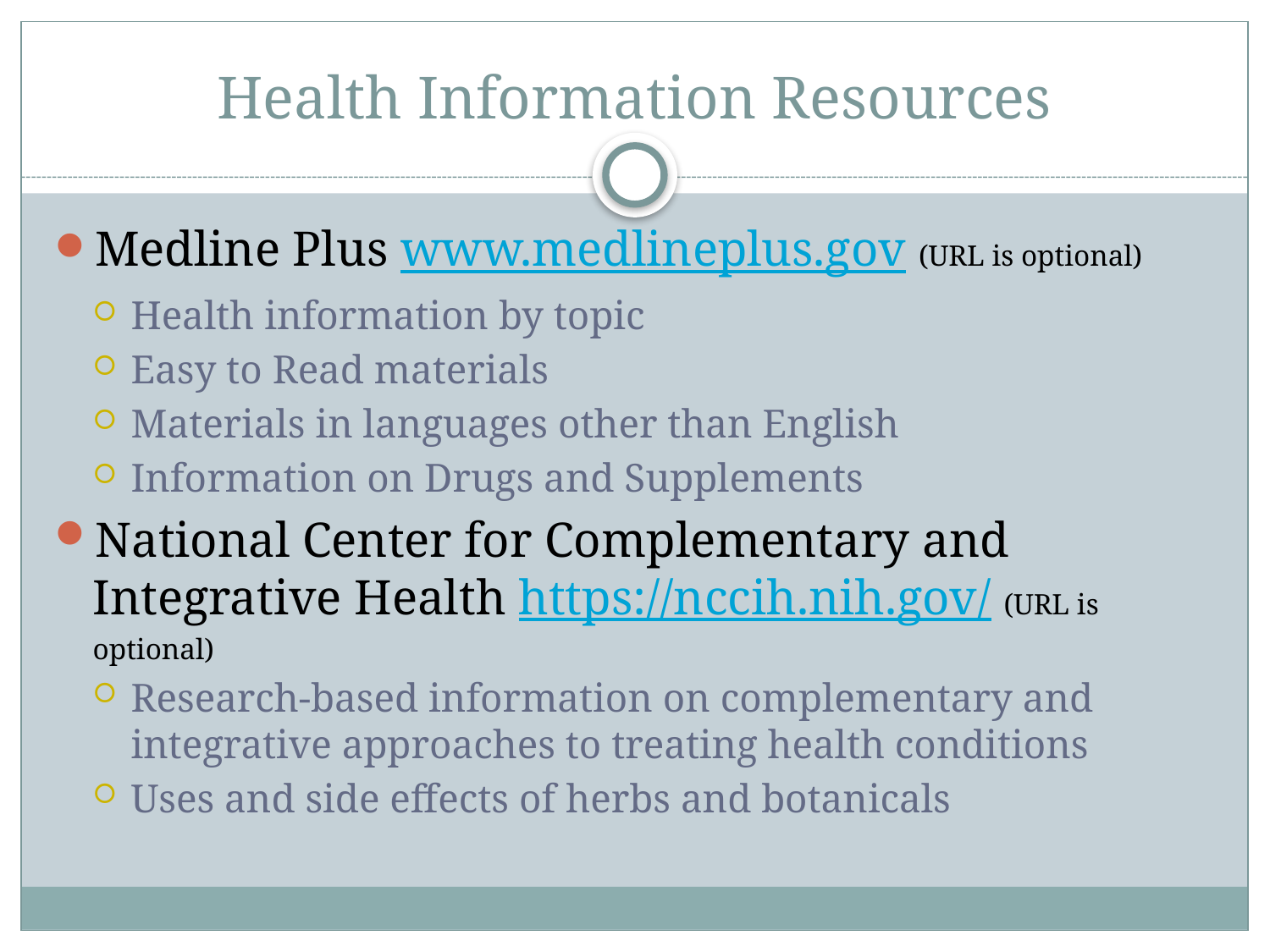

# Health Information Resources
Medline Plus www.medlineplus.gov (URL is optional)
Health information by topic
Easy to Read materials
Materials in languages other than English
Information on Drugs and Supplements
National Center for Complementary and Integrative Health https://nccih.nih.gov/ (URL is optional)
Research-based information on complementary and integrative approaches to treating health conditions
Uses and side effects of herbs and botanicals

## Slide 20
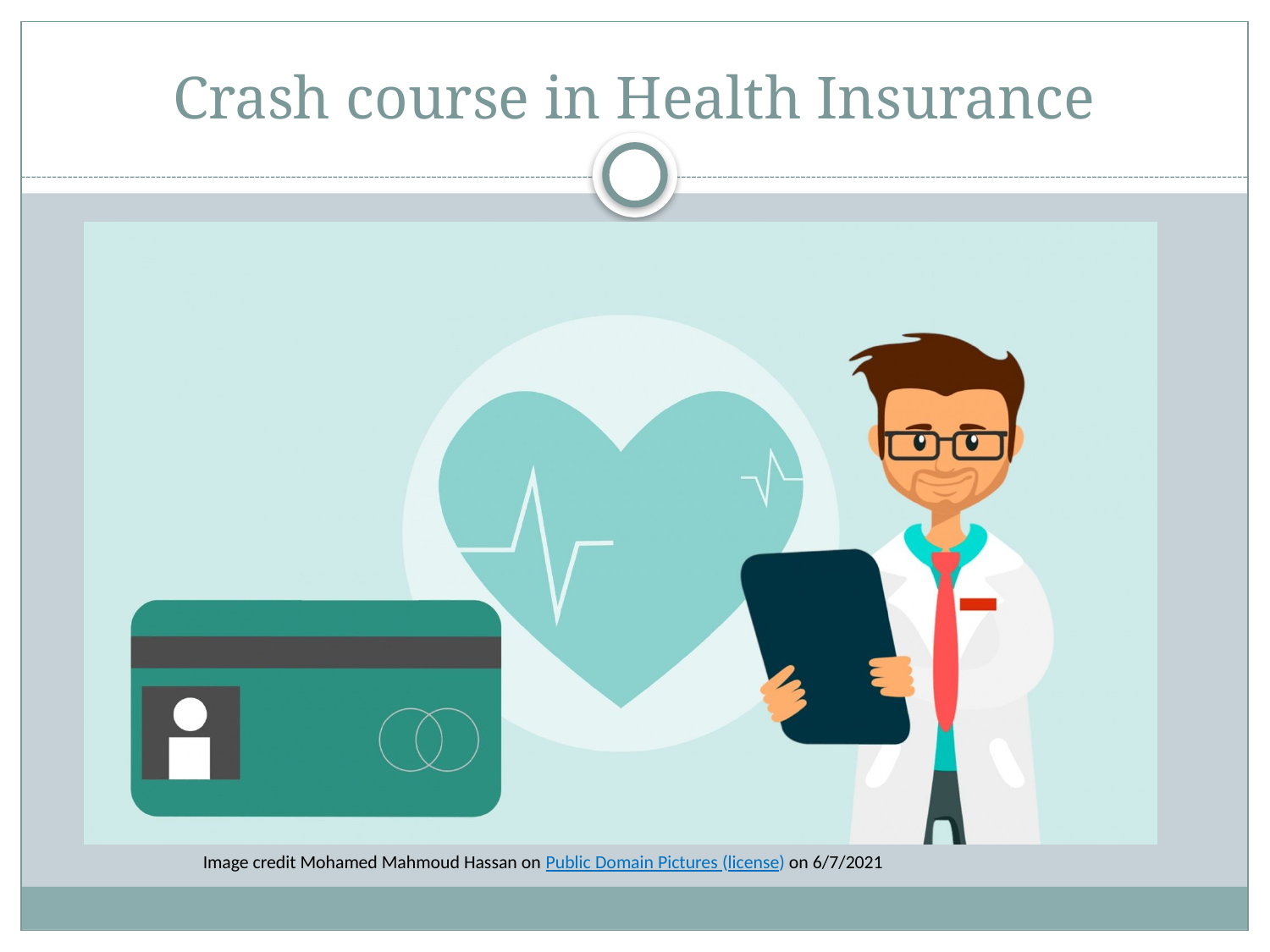

# Crash course in Health Insurance
Image credit Mohamed Mahmoud Hassan on Public Domain Pictures (license) on 6/7/2021

## Slide 21
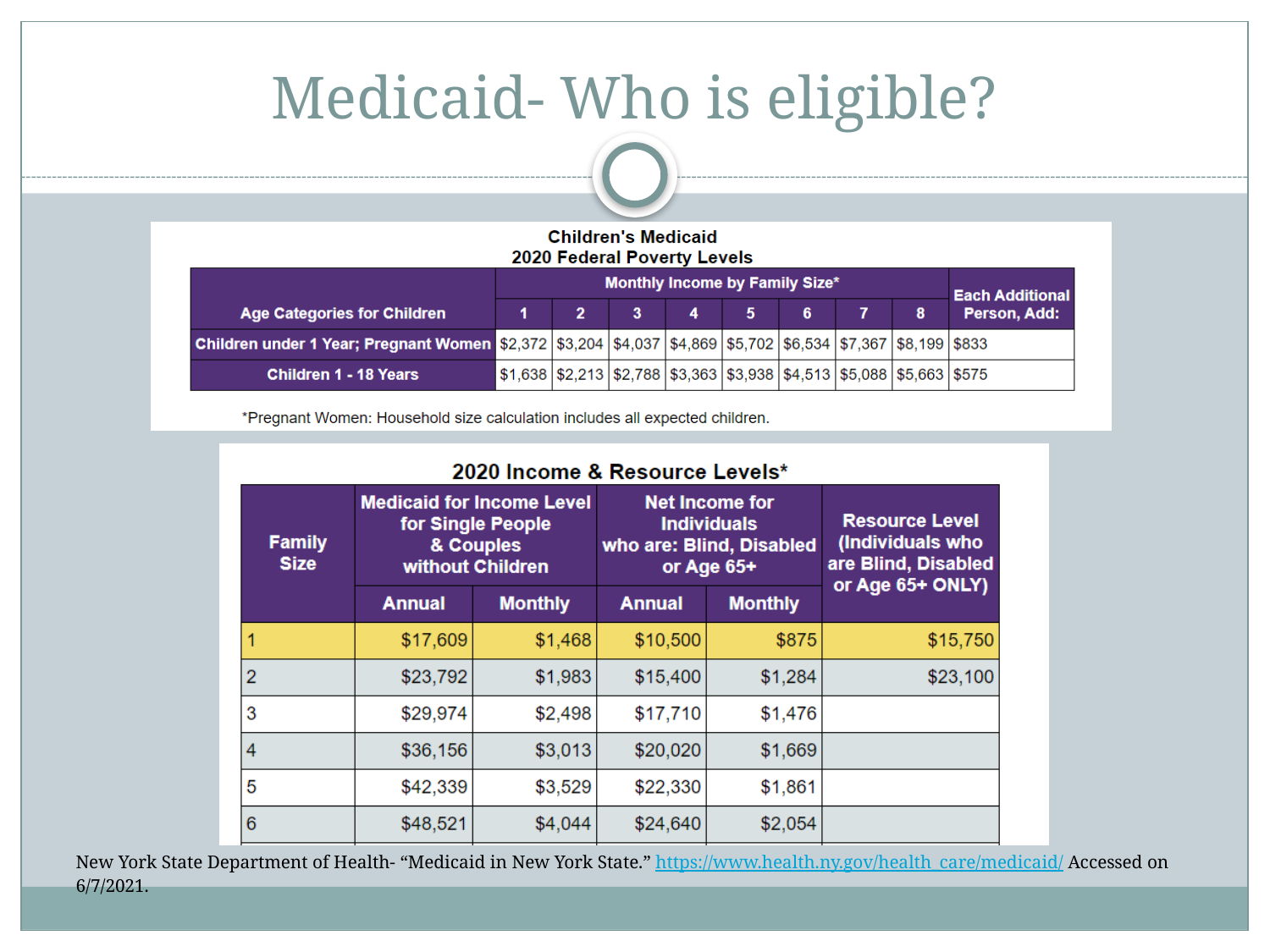

# Medicaid- Who is eligible?
New York State Department of Health- “Medicaid in New York State.” https://www.health.ny.gov/health_care/medicaid/ Accessed on 6/7/2021.

## Slide 22
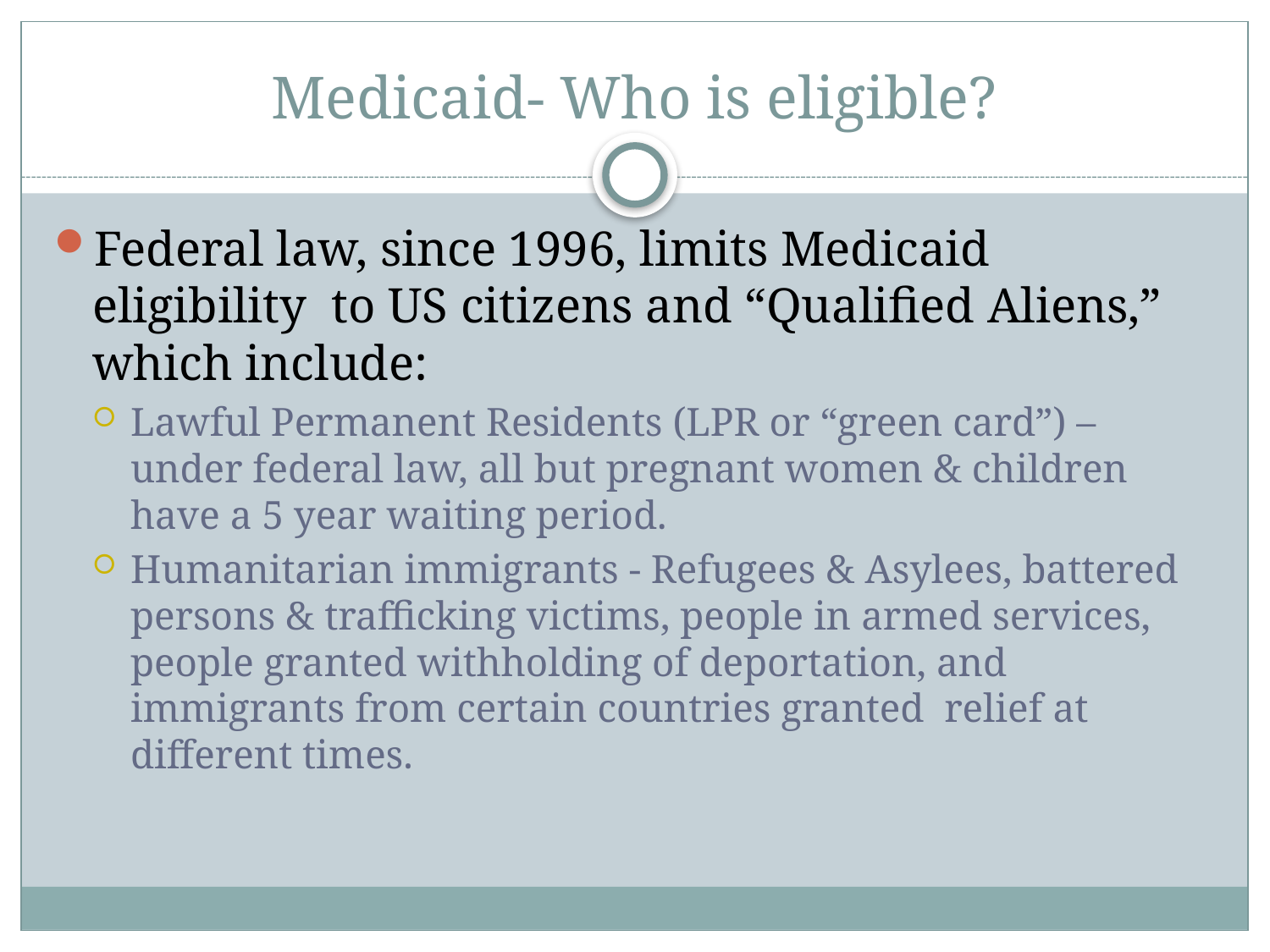

# Medicaid- Who is eligible?
Federal law, since 1996, limits Medicaid eligibility  to US citizens and “Qualified Aliens,” which include:
Lawful Permanent Residents (LPR or “green card”) – under federal law, all but pregnant women & children have a 5 year waiting period.
Humanitarian immigrants - Refugees & Asylees, battered persons & trafficking victims, people in armed services, people granted withholding of deportation, and immigrants from certain countries granted  relief at different times.

## Slide 23
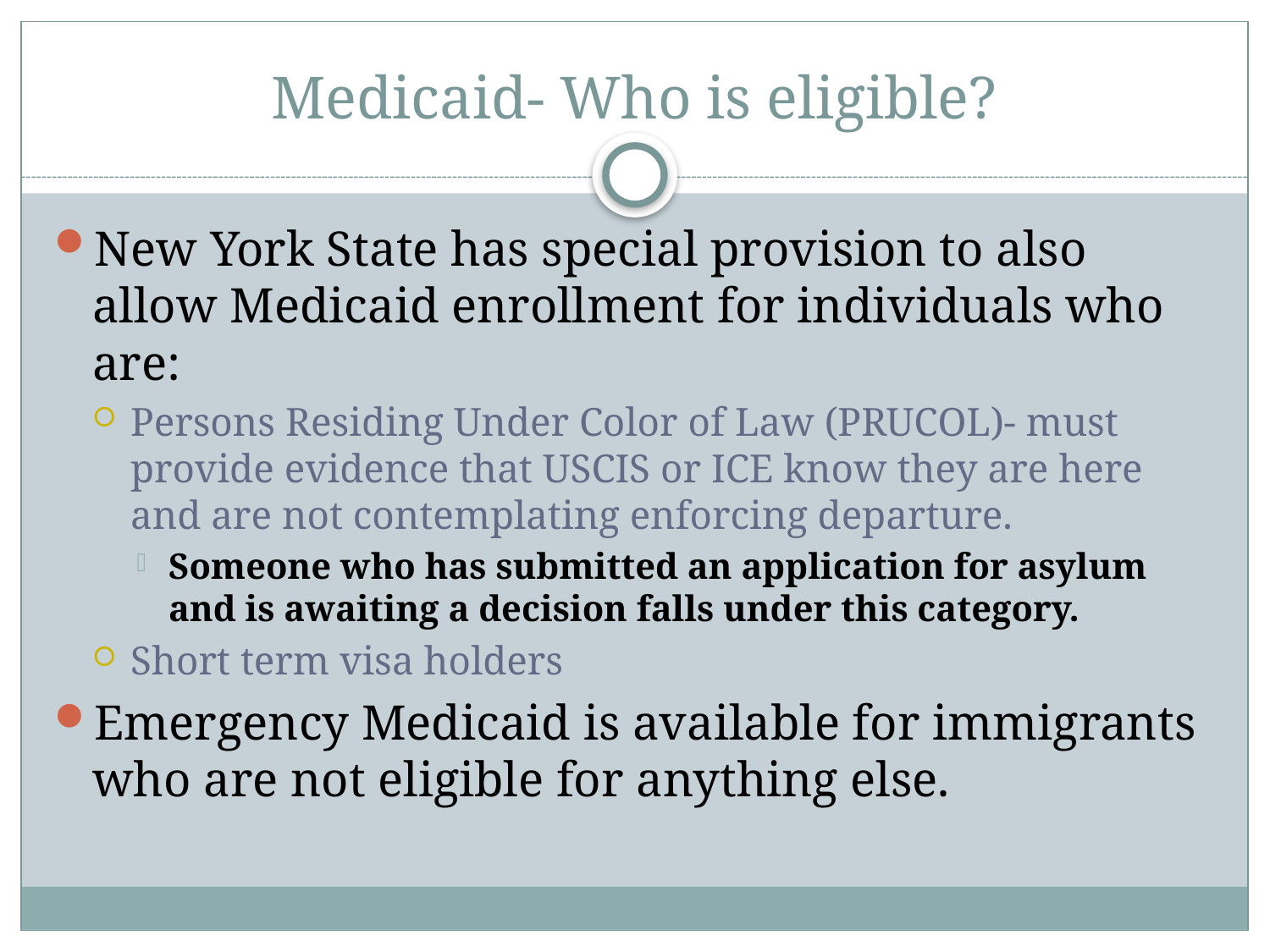

# Medicaid- Who is eligible?
New York State has special provision to also allow Medicaid enrollment for individuals who are:
Persons Residing Under Color of Law (PRUCOL)- must provide evidence that USCIS or ICE know they are here and are not contemplating enforcing departure.
Someone who has submitted an application for asylum and is awaiting a decision falls under this category.
Short term visa holders
Emergency Medicaid is available for immigrants who are not eligible for anything else.

## Slide 24
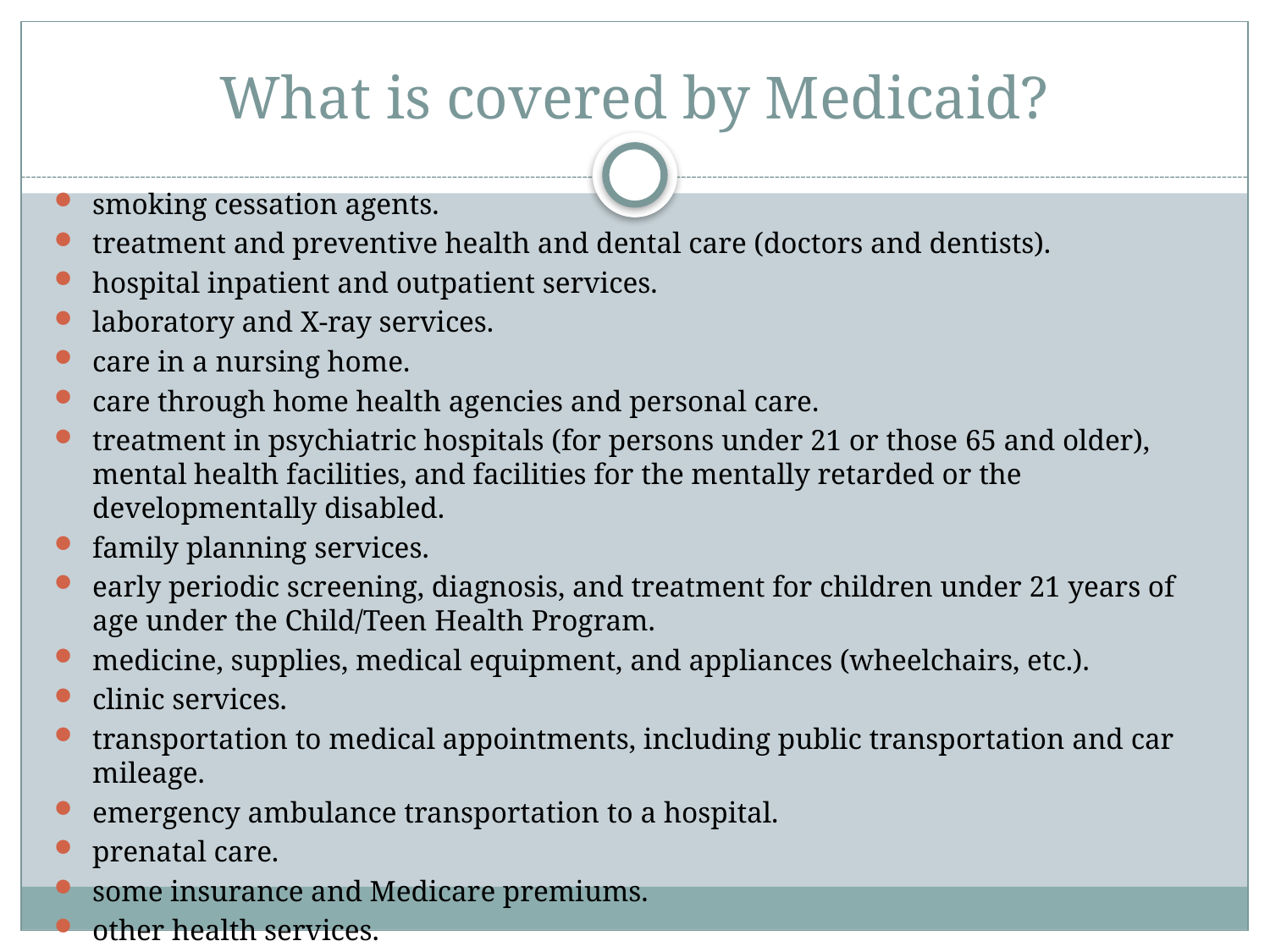

# What is covered by Medicaid?
smoking cessation agents.
treatment and preventive health and dental care (doctors and dentists).
hospital inpatient and outpatient services.
laboratory and X-ray services.
care in a nursing home.
care through home health agencies and personal care.
treatment in psychiatric hospitals (for persons under 21 or those 65 and older), mental health facilities, and facilities for the mentally retarded or the developmentally disabled.
family planning services.
early periodic screening, diagnosis, and treatment for children under 21 years of age under the Child/Teen Health Program.
medicine, supplies, medical equipment, and appliances (wheelchairs, etc.).
clinic services.
transportation to medical appointments, including public transportation and car mileage.
emergency ambulance transportation to a hospital.
prenatal care.
some insurance and Medicare premiums.
other health services.

## Slide 25
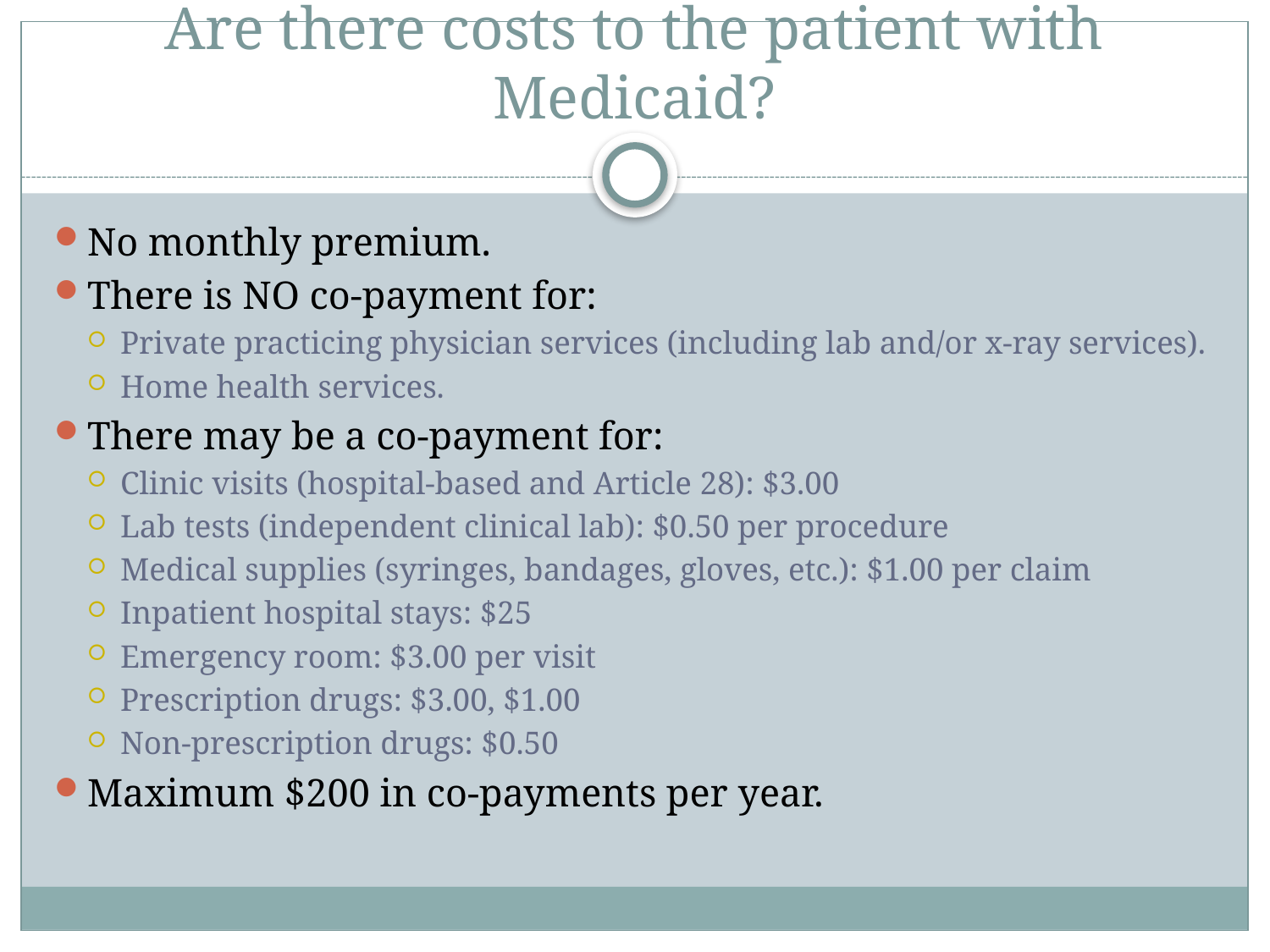

# Are there costs to the patient with Medicaid?
No monthly premium.
There is NO co-payment for:
Private practicing physician services (including lab and/or x-ray services).
Home health services.
There may be a co-payment for:
Clinic visits (hospital-based and Article 28): $3.00
Lab tests (independent clinical lab): $0.50 per procedure
Medical supplies (syringes, bandages, gloves, etc.): $1.00 per claim
Inpatient hospital stays: $25
Emergency room: $3.00 per visit
Prescription drugs: $3.00, $1.00
Non-prescription drugs: $0.50
Maximum $200 in co-payments per year.

## Slide 26
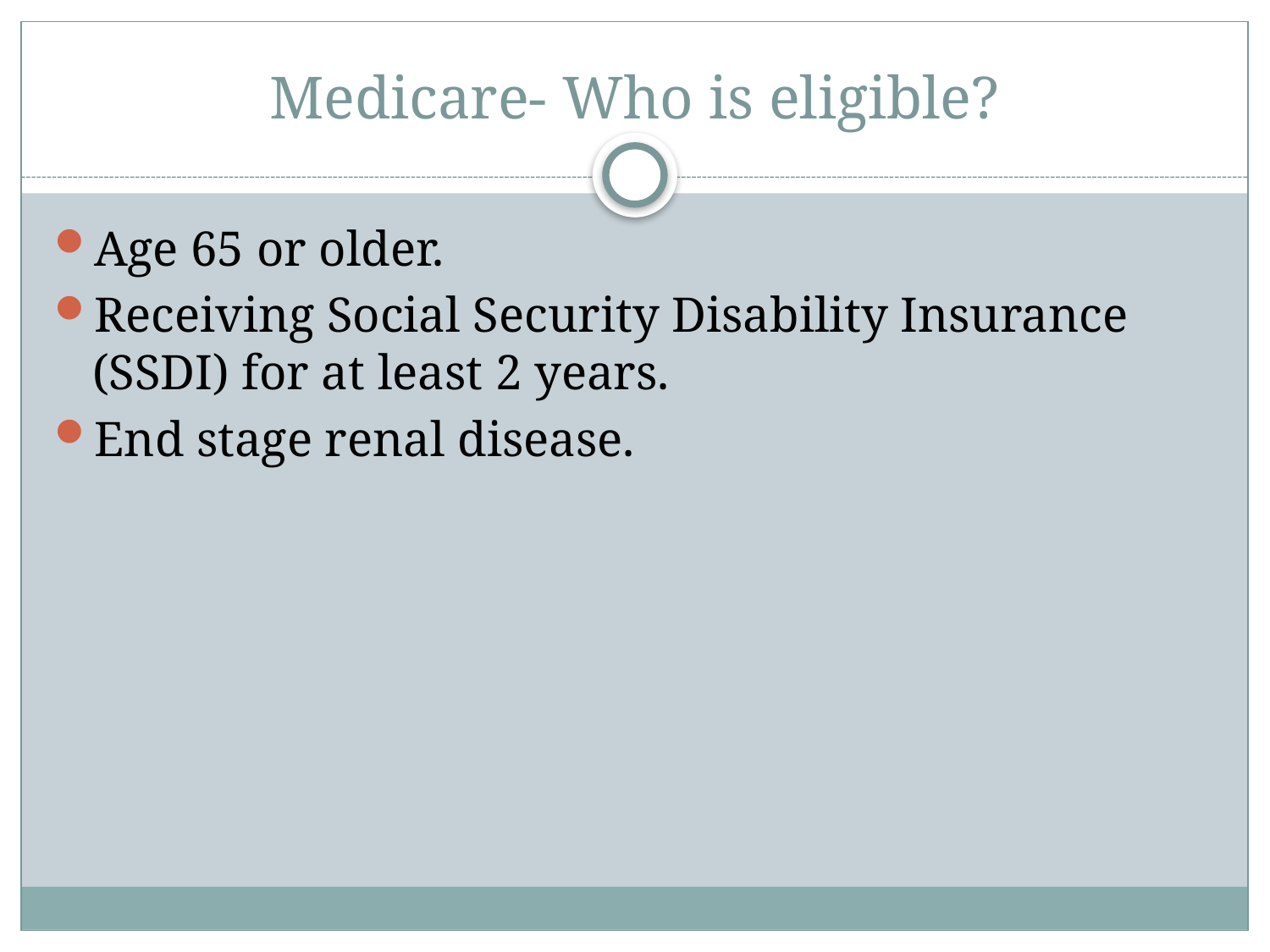

# Medicare- Who is eligible?
Age 65 or older.
Receiving Social Security Disability Insurance (SSDI) for at least 2 years.
End stage renal disease.

## Slide 27
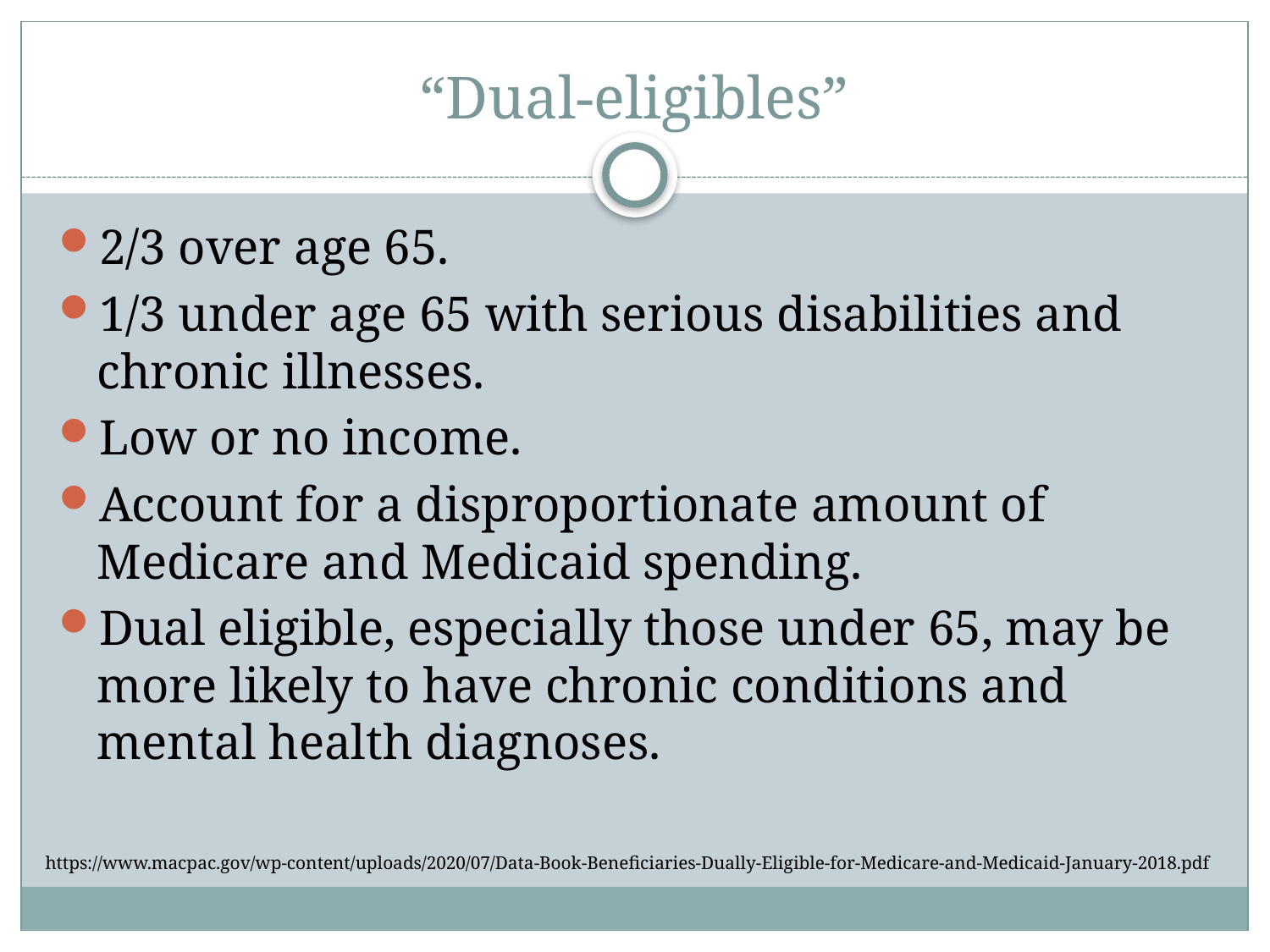

# “Dual-eligibles”
2/3 over age 65.
1/3 under age 65 with serious disabilities and chronic illnesses.
Low or no income.
Account for a disproportionate amount of Medicare and Medicaid spending.
Dual eligible, especially those under 65, may be more likely to have chronic conditions and mental health diagnoses.
https://www.macpac.gov/wp-content/uploads/2020/07/Data-Book-Beneficiaries-Dually-Eligible-for-Medicare-and-Medicaid-January-2018.pdf

## Slide 28
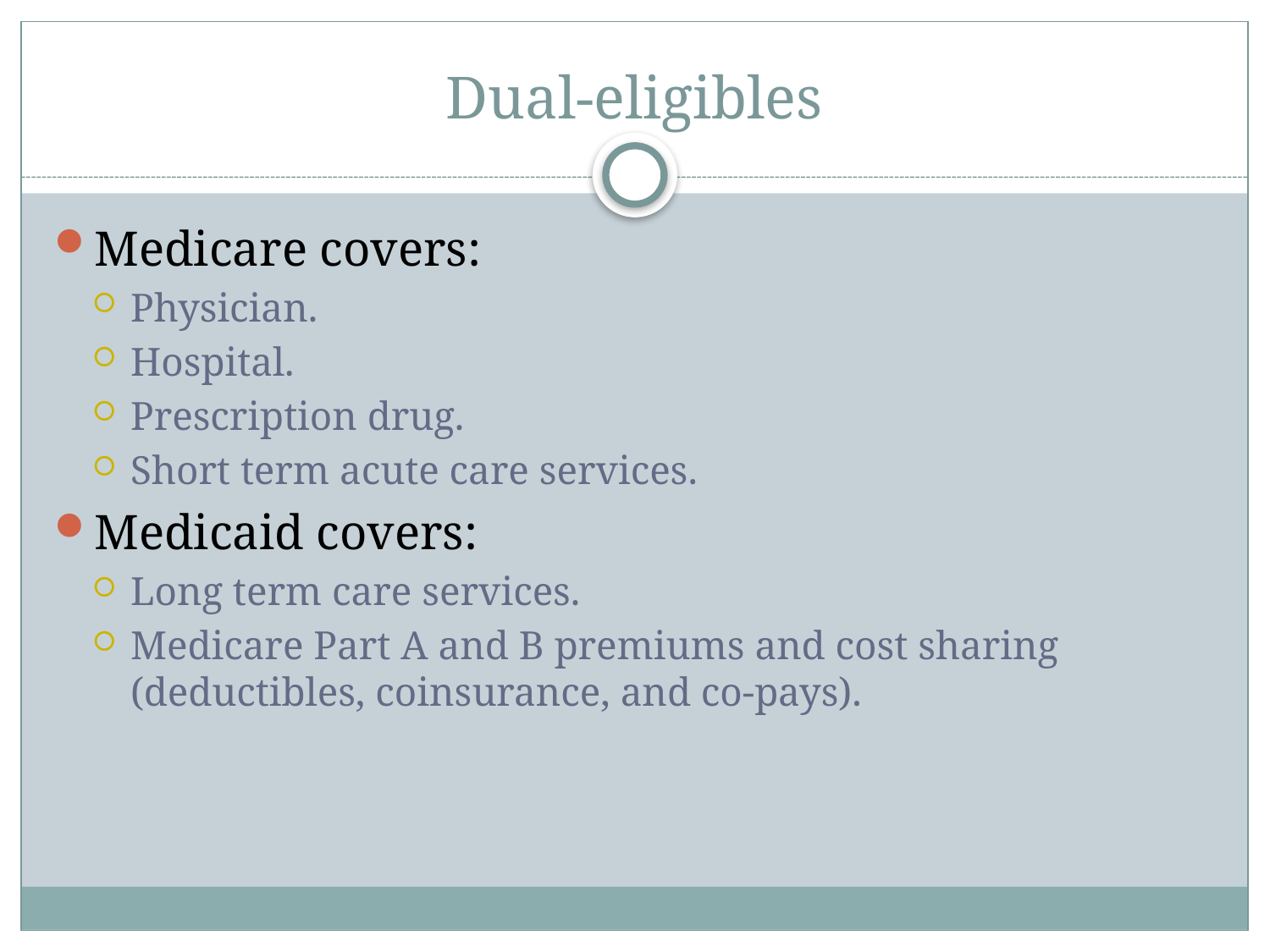

# Dual-eligibles
Medicare covers:
Physician.
Hospital.
Prescription drug.
Short term acute care services.
Medicaid covers:
Long term care services.
Medicare Part A and B premiums and cost sharing (deductibles, coinsurance, and co-pays).

## Slide 29
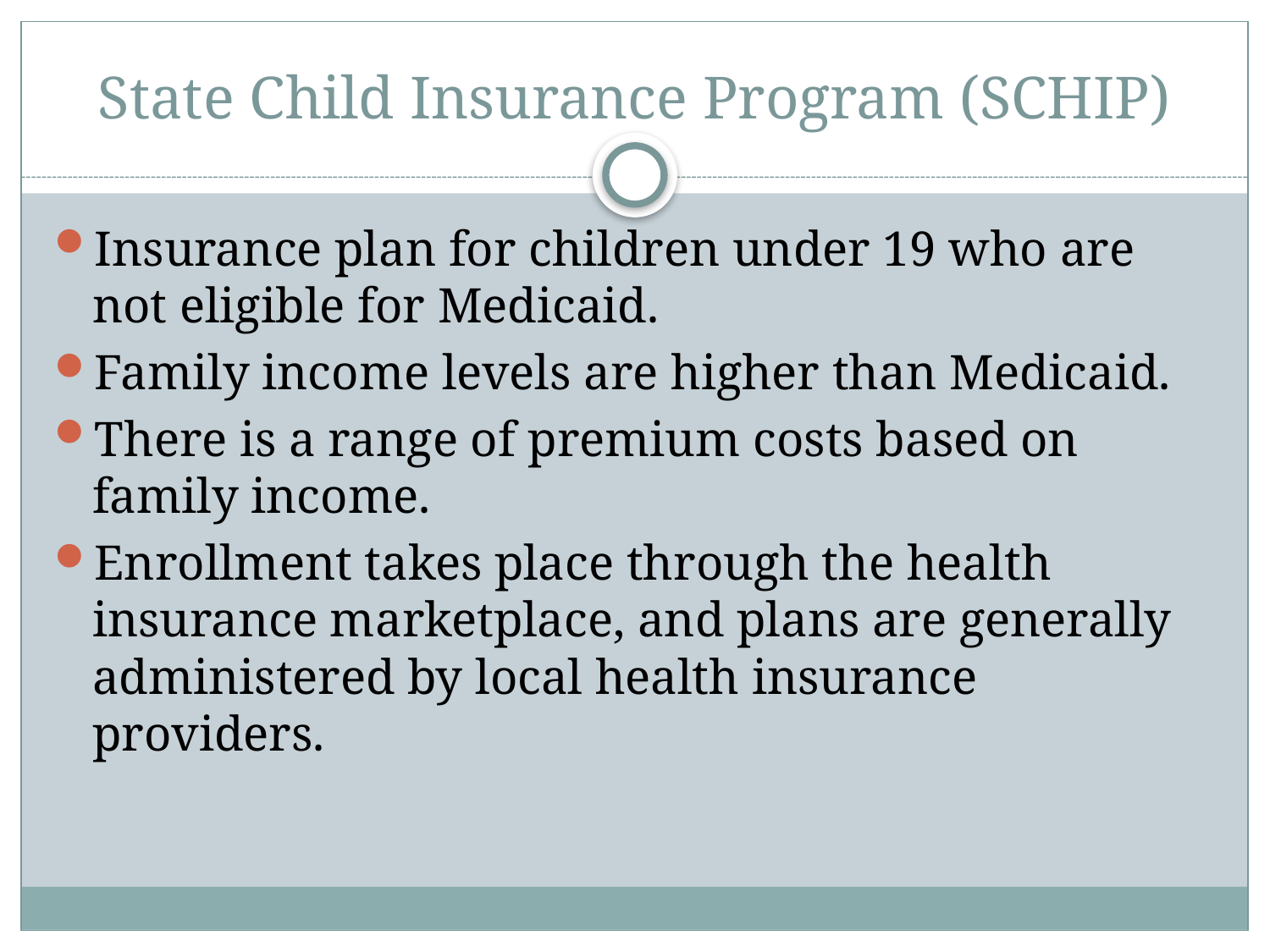

# State Child Insurance Program (SCHIP)
Insurance plan for children under 19 who are not eligible for Medicaid.
Family income levels are higher than Medicaid.
There is a range of premium costs based on family income.
Enrollment takes place through the health insurance marketplace, and plans are generally administered by local health insurance providers.

## Slide 30
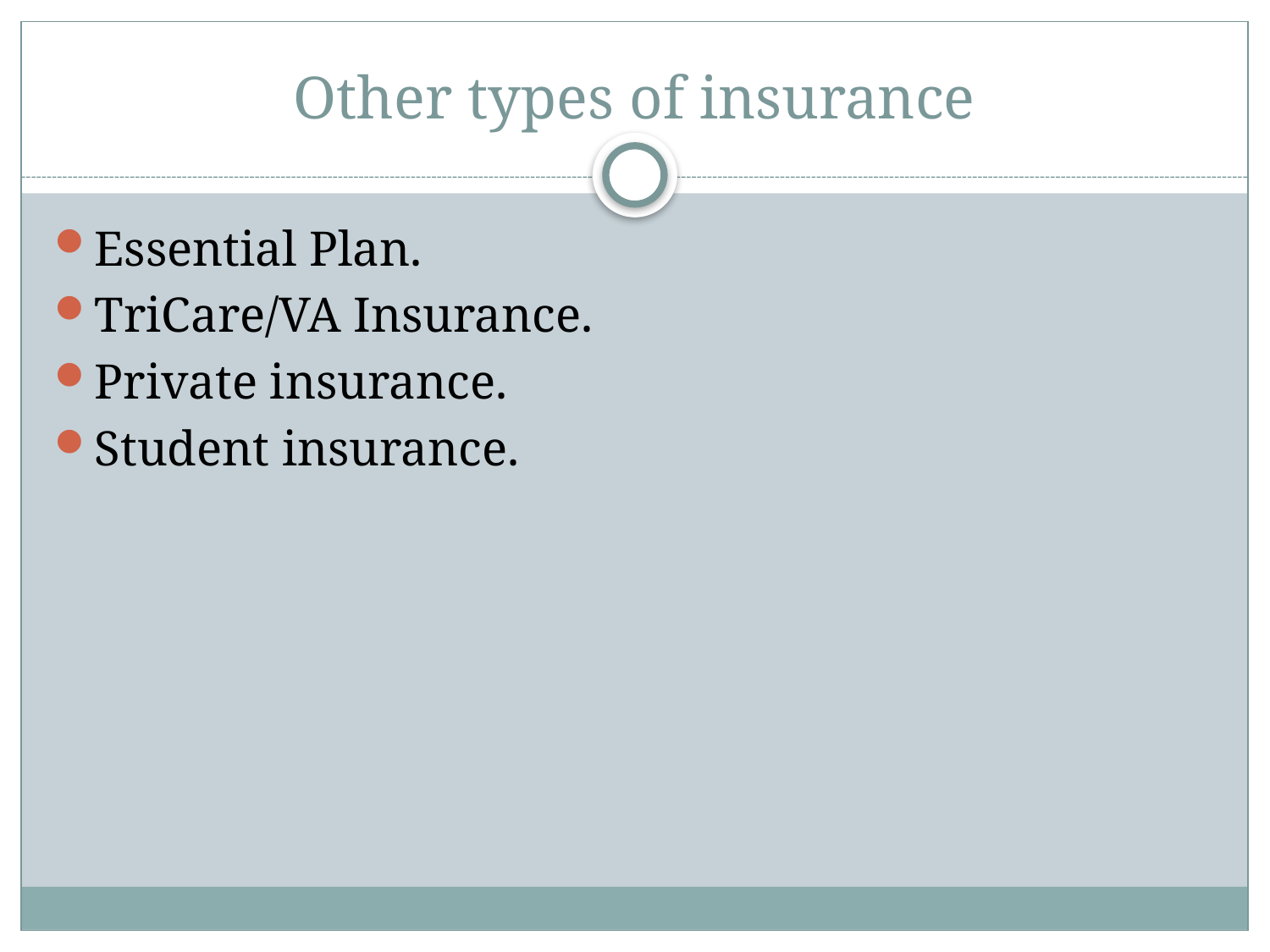

# Other types of insurance
Essential Plan.
TriCare/VA Insurance.
Private insurance.
Student insurance.

## Slide 31
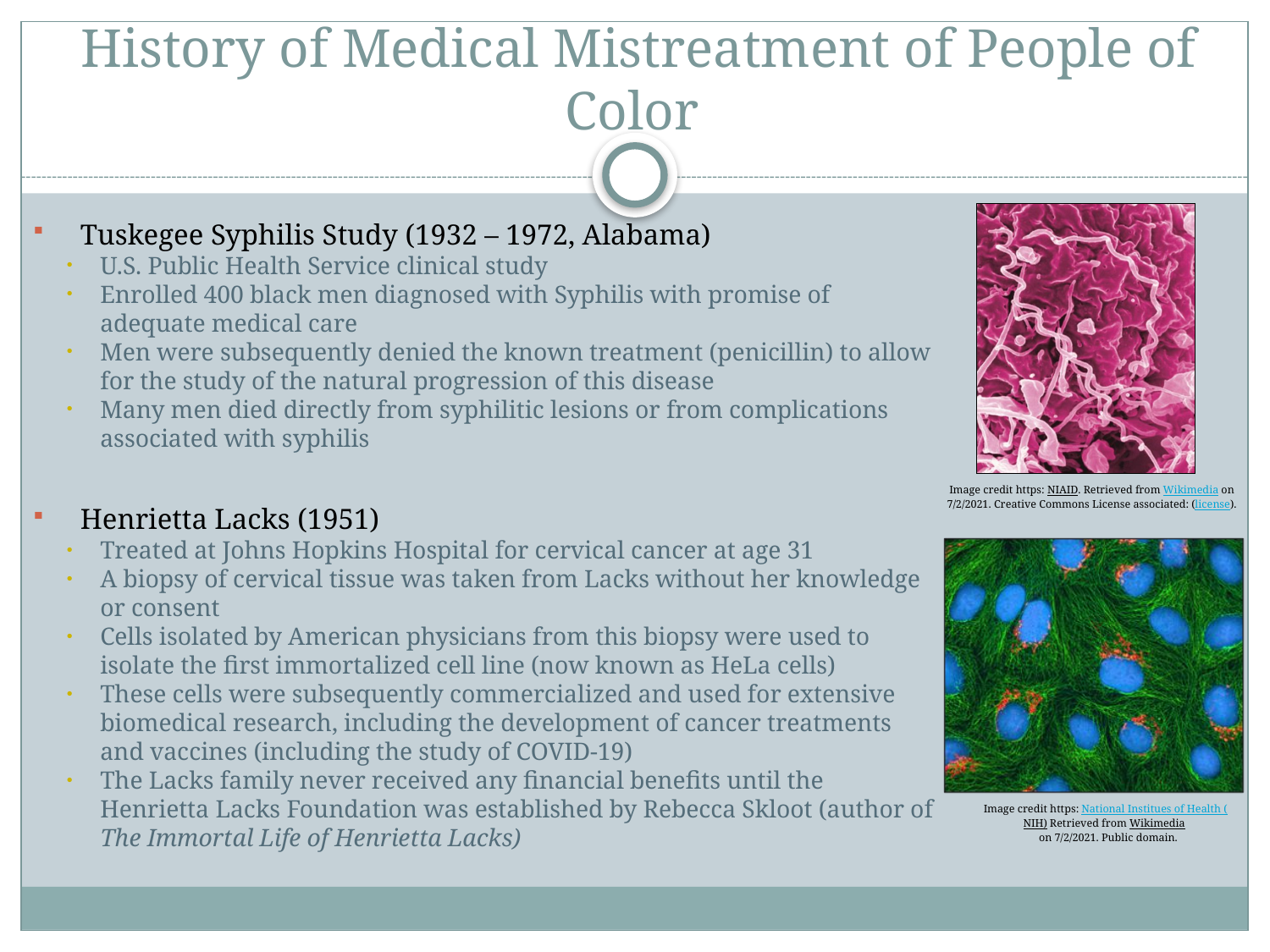

# History of Medical Mistreatment of People of Color
Tuskegee Syphilis Study (1932 – 1972, Alabama)
U.S. Public Health Service clinical study
Enrolled 400 black men diagnosed with Syphilis with promise of adequate medical care
Men were subsequently denied the known treatment (penicillin) to allow for the study of the natural progression of this disease
Many men died directly from syphilitic lesions or from complications associated with syphilis
Henrietta Lacks (1951)
Treated at Johns Hopkins Hospital for cervical cancer at age 31
A biopsy of cervical tissue was taken from Lacks without her knowledge or consent
Cells isolated by American physicians from this biopsy were used to isolate the first immortalized cell line (now known as HeLa cells)
These cells were subsequently commercialized and used for extensive biomedical research, including the development of cancer treatments and vaccines (including the study of COVID-19)
The Lacks family never received any financial benefits until the Henrietta Lacks Foundation was established by Rebecca Skloot (author of The Immortal Life of Henrietta Lacks)
Image credit https: NIAID. Retrieved from Wikimedia on 7/2/2021. Creative Commons License associated: (license).
Image credit https: National Institues of Health (NIH) Retrieved from Wikimedia 
  on 7/2/2021. Public domain.

## Slide 32
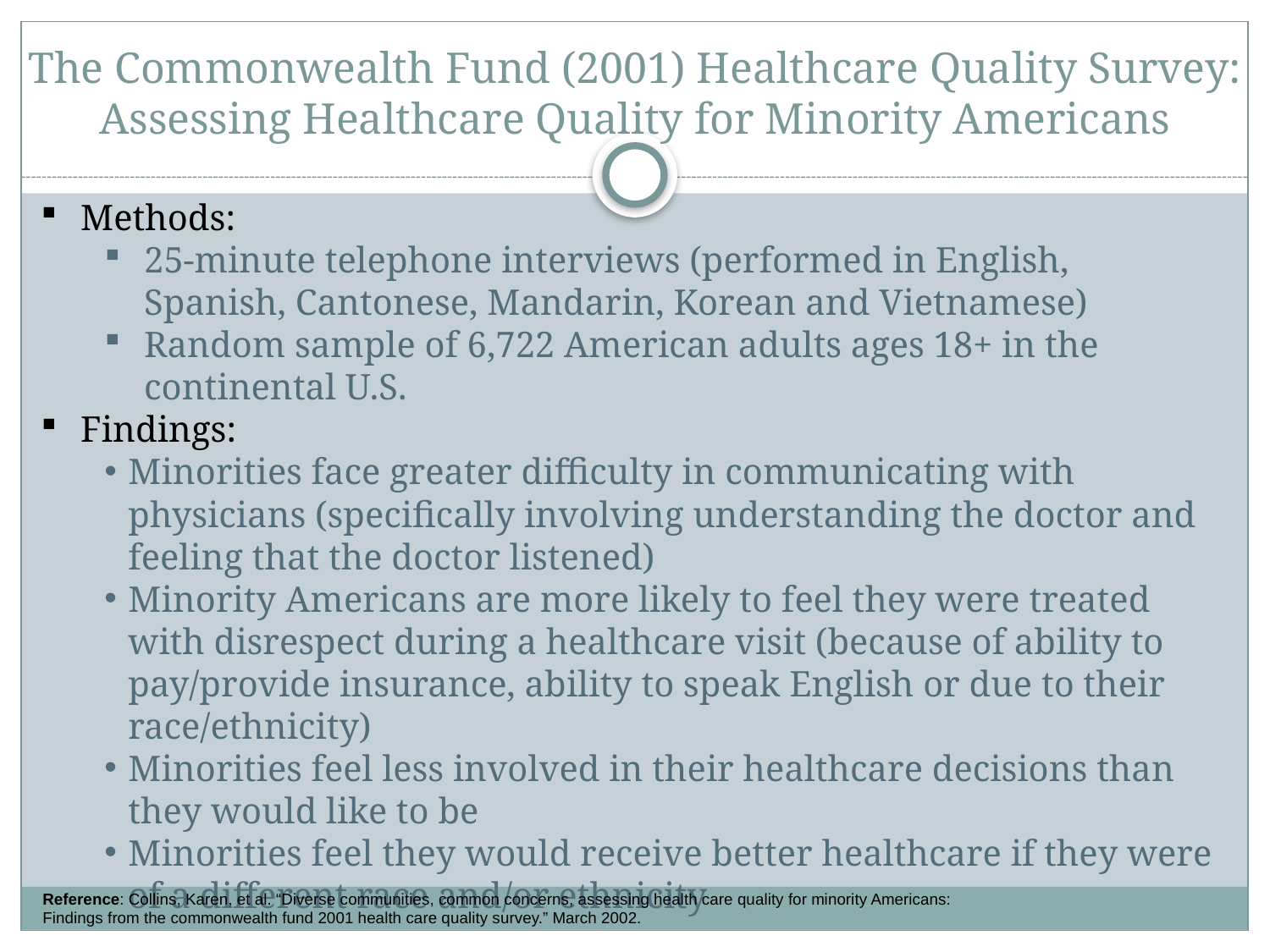

# The Commonwealth Fund (2001) Healthcare Quality Survey: Assessing Healthcare Quality for Minority Americans
Methods:
25-minute telephone interviews (performed in English, Spanish, Cantonese, Mandarin, Korean and Vietnamese)
Random sample of 6,722 American adults ages 18+ in the continental U.S.
Findings:
Minorities face greater difficulty in communicating with physicians (specifically involving understanding the doctor and feeling that the doctor listened)
Minority Americans are more likely to feel they were treated with disrespect during a healthcare visit (because of ability to pay/provide insurance, ability to speak English or due to their race/ethnicity)
Minorities feel less involved in their healthcare decisions than they would like to be
Minorities feel they would receive better healthcare if they were of a different race and/or ethnicity
Reference: Collins, Karen, et al. “Diverse communities, common concerns, assessing health care quality for minority Americans:  Findings from the commonwealth fund 2001 health care quality survey.” March 2002.

## Slide 33
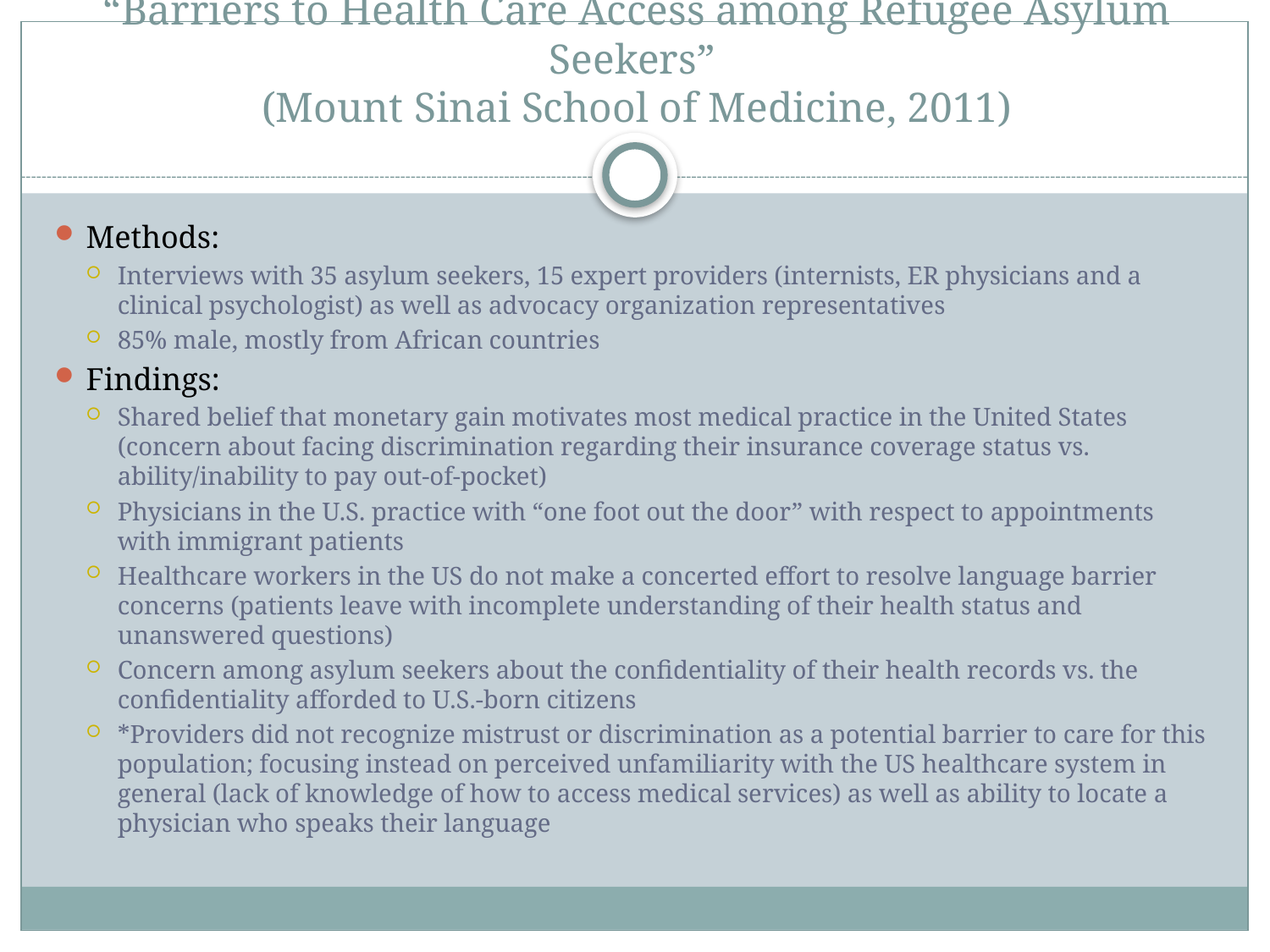

# “Barriers to Health Care Access among Refugee Asylum Seekers” (Mount Sinai School of Medicine, 2011)
Methods:
Interviews with 35 asylum seekers, 15 expert providers (internists, ER physicians and a clinical psychologist) as well as advocacy organization representatives
85% male, mostly from African countries
Findings:
Shared belief that monetary gain motivates most medical practice in the United States (concern about facing discrimination regarding their insurance coverage status vs. ability/inability to pay out-of-pocket)
Physicians in the U.S. practice with “one foot out the door” with respect to appointments with immigrant patients
Healthcare workers in the US do not make a concerted effort to resolve language barrier concerns (patients leave with incomplete understanding of their health status and unanswered questions)
Concern among asylum seekers about the confidentiality of their health records vs. the confidentiality afforded to U.S.-born citizens
*Providers did not recognize mistrust or discrimination as a potential barrier to care for this population; focusing instead on perceived unfamiliarity with the US healthcare system in general (lack of knowledge of how to access medical services) as well as ability to locate a physician who speaks their language

## Slide 34
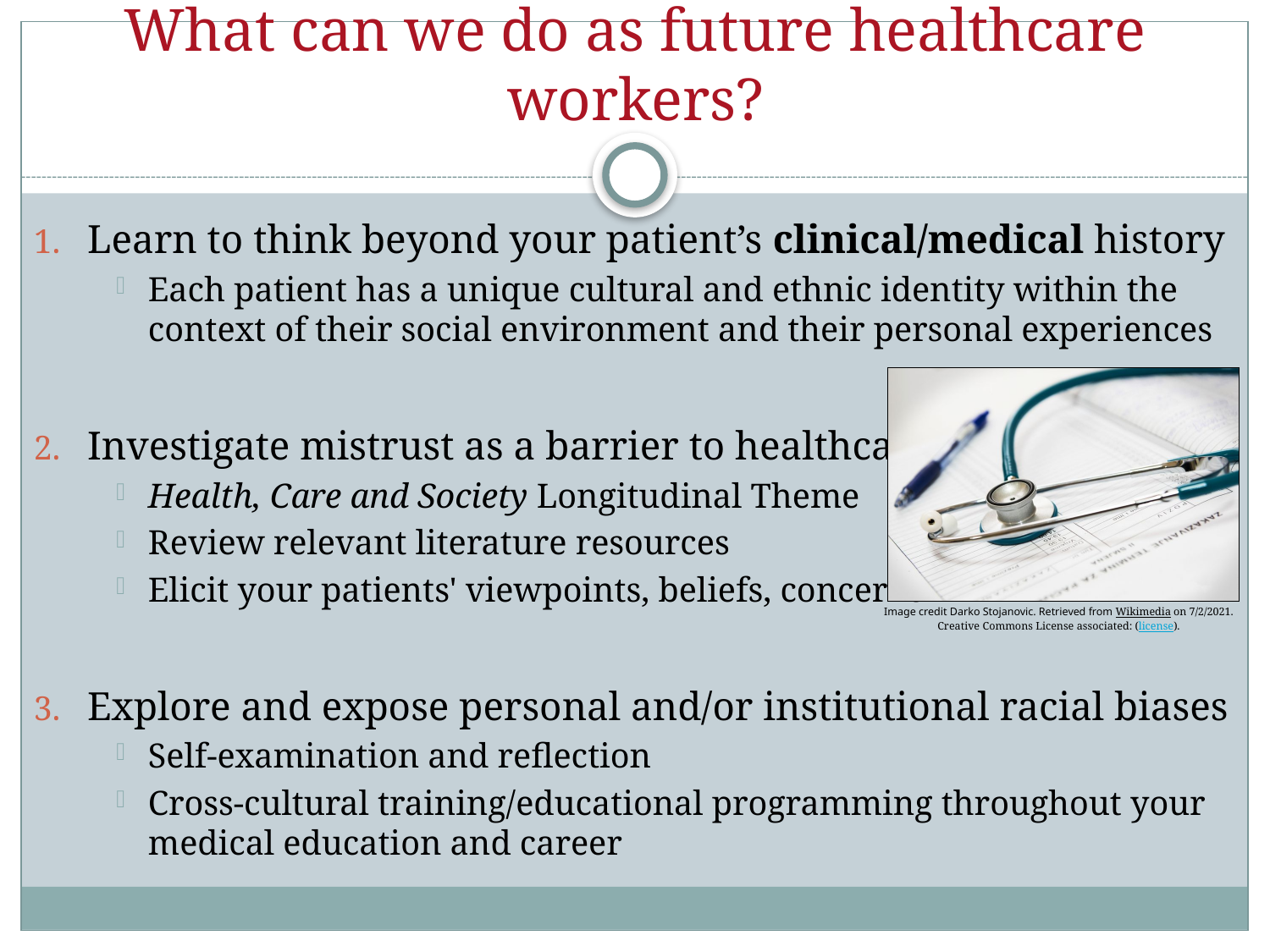

# What can we do as future healthcare workers?
Learn to think beyond your patient’s clinical/medical history
Each patient has a unique cultural and ethnic identity within the context of their social environment and their personal experiences
Investigate mistrust as a barrier to healthcare
Health, Care and Society Longitudinal Theme
Review relevant literature resources
Elicit your patients' viewpoints, beliefs, concerns
Explore and expose personal and/or institutional racial biases
Self-examination and reflection
Cross-cultural training/educational programming throughout your medical education and career
Image credit Darko Stojanovic. Retrieved from Wikimedia on 7/2/2021. Creative Commons License associated: (license).

## Slide 35
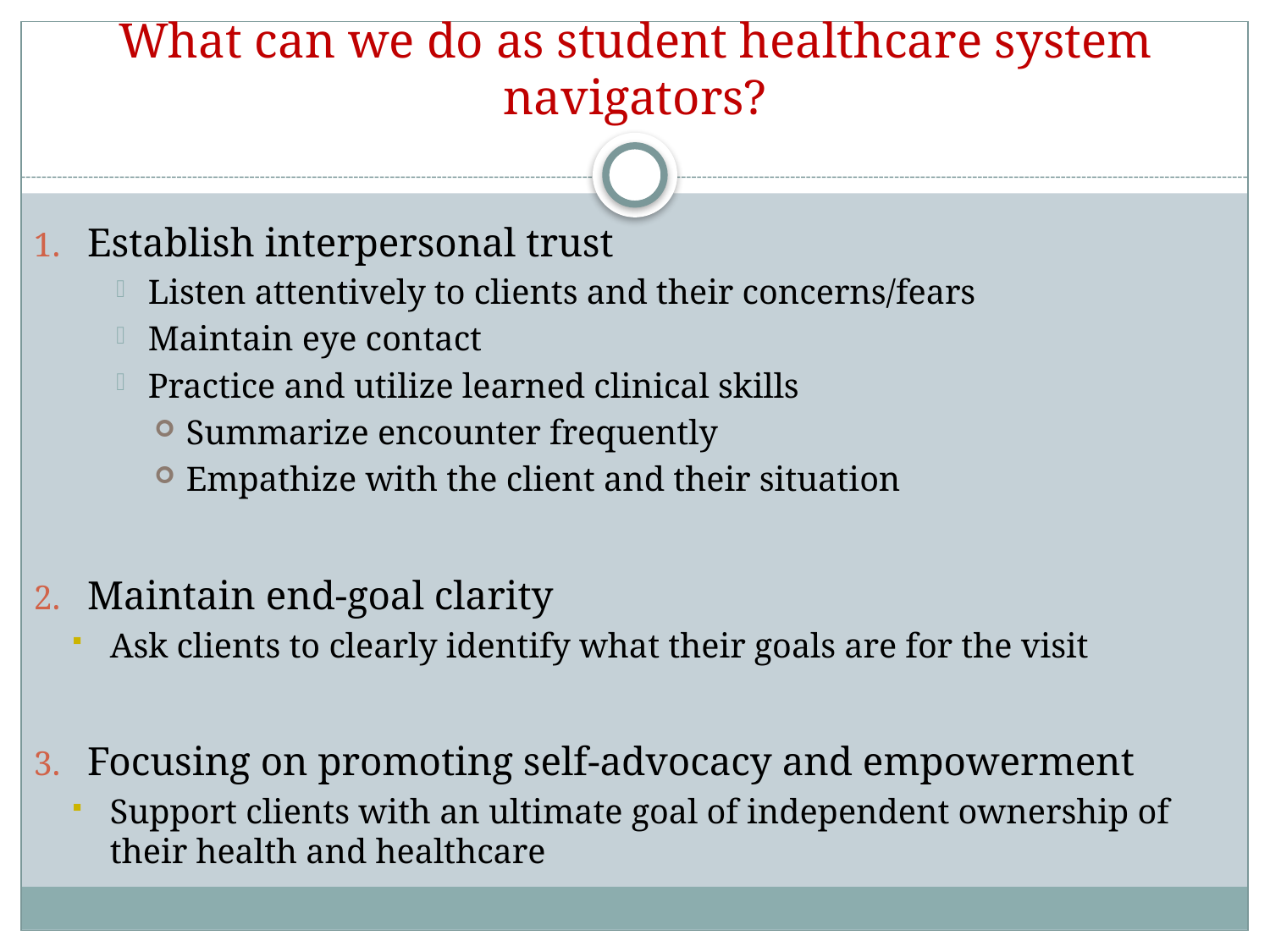

# What can we do as student healthcare system navigators?
Establish interpersonal trust
Listen attentively to clients and their concerns/fears
Maintain eye contact
Practice and utilize learned clinical skills
Summarize encounter frequently
Empathize with the client and their situation
Maintain end-goal clarity
Ask clients to clearly identify what their goals are for the visit
Focusing on promoting self-advocacy and empowerment
Support clients with an ultimate goal of independent ownership of their health and healthcare

## Slide 36
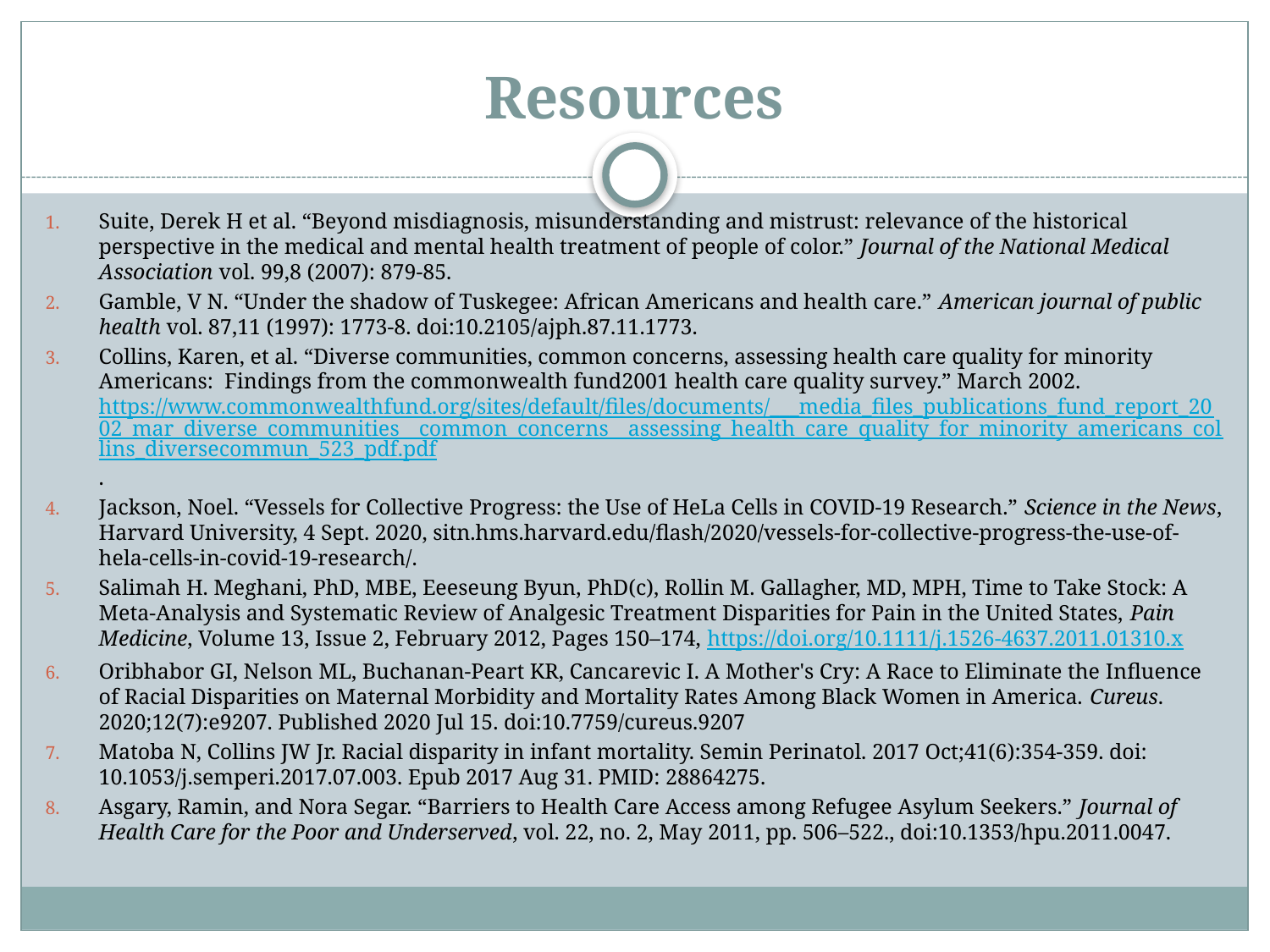

# Resources
Suite, Derek H et al. “Beyond misdiagnosis, misunderstanding and mistrust: relevance of the historical perspective in the medical and mental health treatment of people of color.” Journal of the National Medical Association vol. 99,8 (2007): 879-85.
Gamble, V N. “Under the shadow of Tuskegee: African Americans and health care.” American journal of public health vol. 87,11 (1997): 1773-8. doi:10.2105/ajph.87.11.1773.
Collins, Karen, et al. “Diverse communities, common concerns, assessing health care quality for minority Americans:  Findings from the commonwealth fund2001 health care quality survey.” March 2002. https://www.commonwealthfund.org/sites/default/files/documents/___media_files_publications_fund_report_2002_mar_diverse_communities__common_concerns__assessing_health_care_quality_for_minority_americans_collins_diversecommun_523_pdf.pdf.
Jackson, Noel. “Vessels for Collective Progress: the Use of HeLa Cells in COVID-19 Research.” Science in the News, Harvard University, 4 Sept. 2020, sitn.hms.harvard.edu/flash/2020/vessels-for-collective-progress-the-use-of-hela-cells-in-covid-19-research/.
Salimah H. Meghani, PhD, MBE, Eeeseung Byun, PhD(c), Rollin M. Gallagher, MD, MPH, Time to Take Stock: A Meta-Analysis and Systematic Review of Analgesic Treatment Disparities for Pain in the United States, Pain Medicine, Volume 13, Issue 2, February 2012, Pages 150–174, https://doi.org/10.1111/j.1526-4637.2011.01310.x
Oribhabor GI, Nelson ML, Buchanan-Peart KR, Cancarevic I. A Mother's Cry: A Race to Eliminate the Influence of Racial Disparities on Maternal Morbidity and Mortality Rates Among Black Women in America. Cureus. 2020;12(7):e9207. Published 2020 Jul 15. doi:10.7759/cureus.9207
Matoba N, Collins JW Jr. Racial disparity in infant mortality. Semin Perinatol. 2017 Oct;41(6):354-359. doi: 10.1053/j.semperi.2017.07.003. Epub 2017 Aug 31. PMID: 28864275.
Asgary, Ramin, and Nora Segar. “Barriers to Health Care Access among Refugee Asylum Seekers.” Journal of Health Care for the Poor and Underserved, vol. 22, no. 2, May 2011, pp. 506–522., doi:10.1353/hpu.2011.0047.

## Slide 37
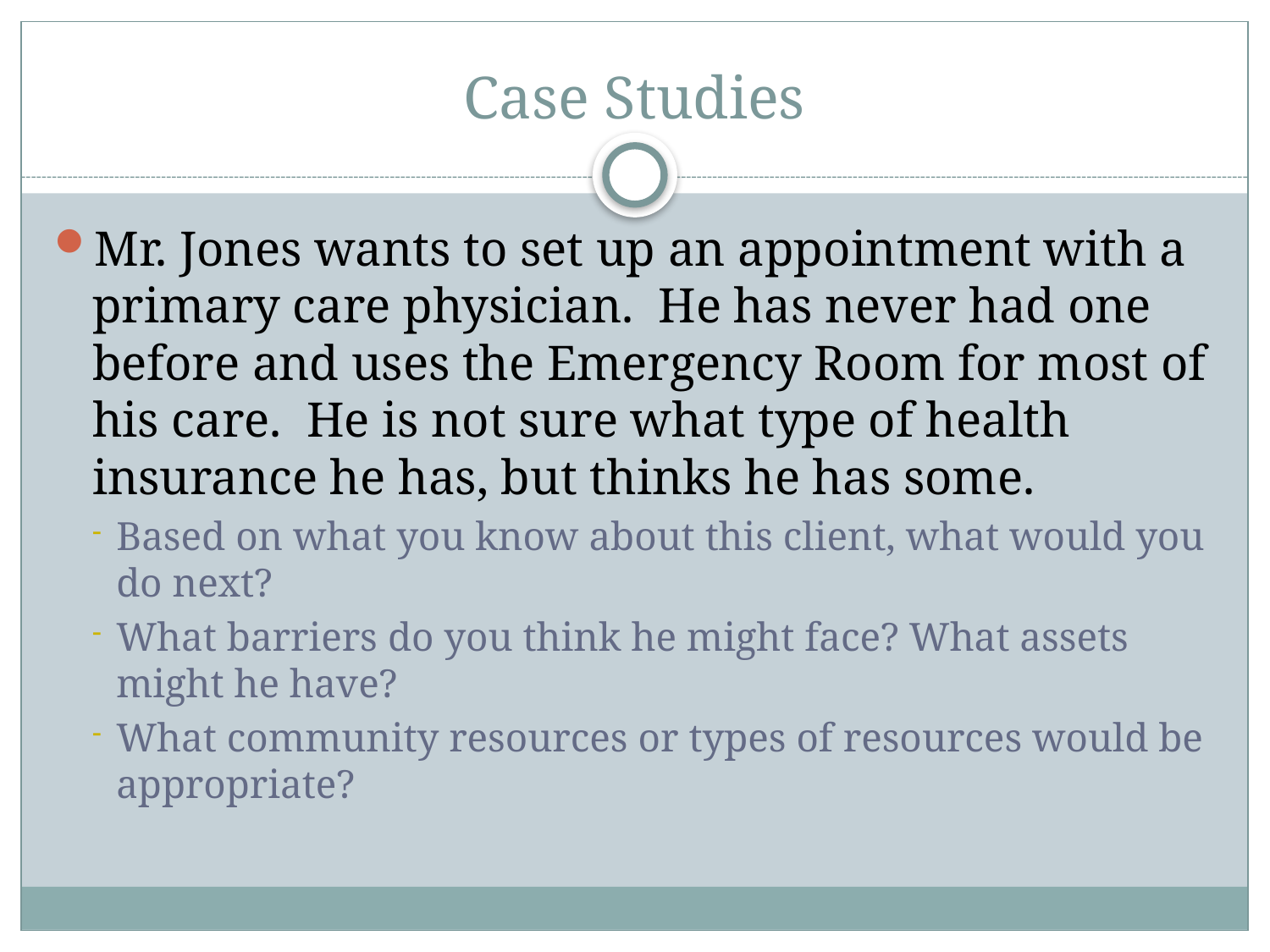

# Case Studies
Mr. Jones wants to set up an appointment with a primary care physician. He has never had one before and uses the Emergency Room for most of his care. He is not sure what type of health insurance he has, but thinks he has some.
Based on what you know about this client, what would you do next?
What barriers do you think he might face? What assets might he have?
What community resources or types of resources would be appropriate?

## Slide 38
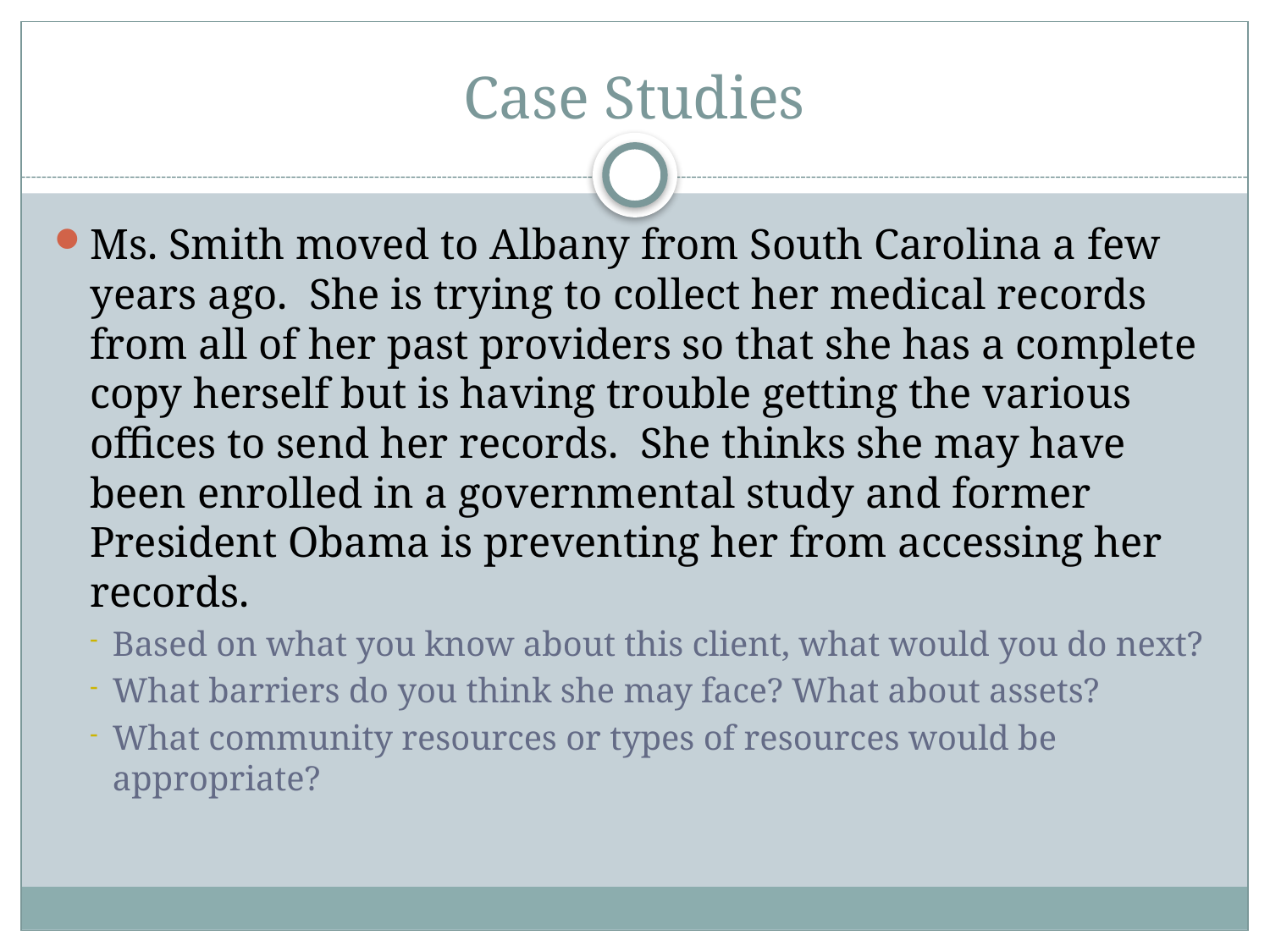

# Case Studies
Ms. Smith moved to Albany from South Carolina a few years ago. She is trying to collect her medical records from all of her past providers so that she has a complete copy herself but is having trouble getting the various offices to send her records. She thinks she may have been enrolled in a governmental study and former President Obama is preventing her from accessing her records.
Based on what you know about this client, what would you do next?
What barriers do you think she may face? What about assets?
What community resources or types of resources would be appropriate?

## Slide 39
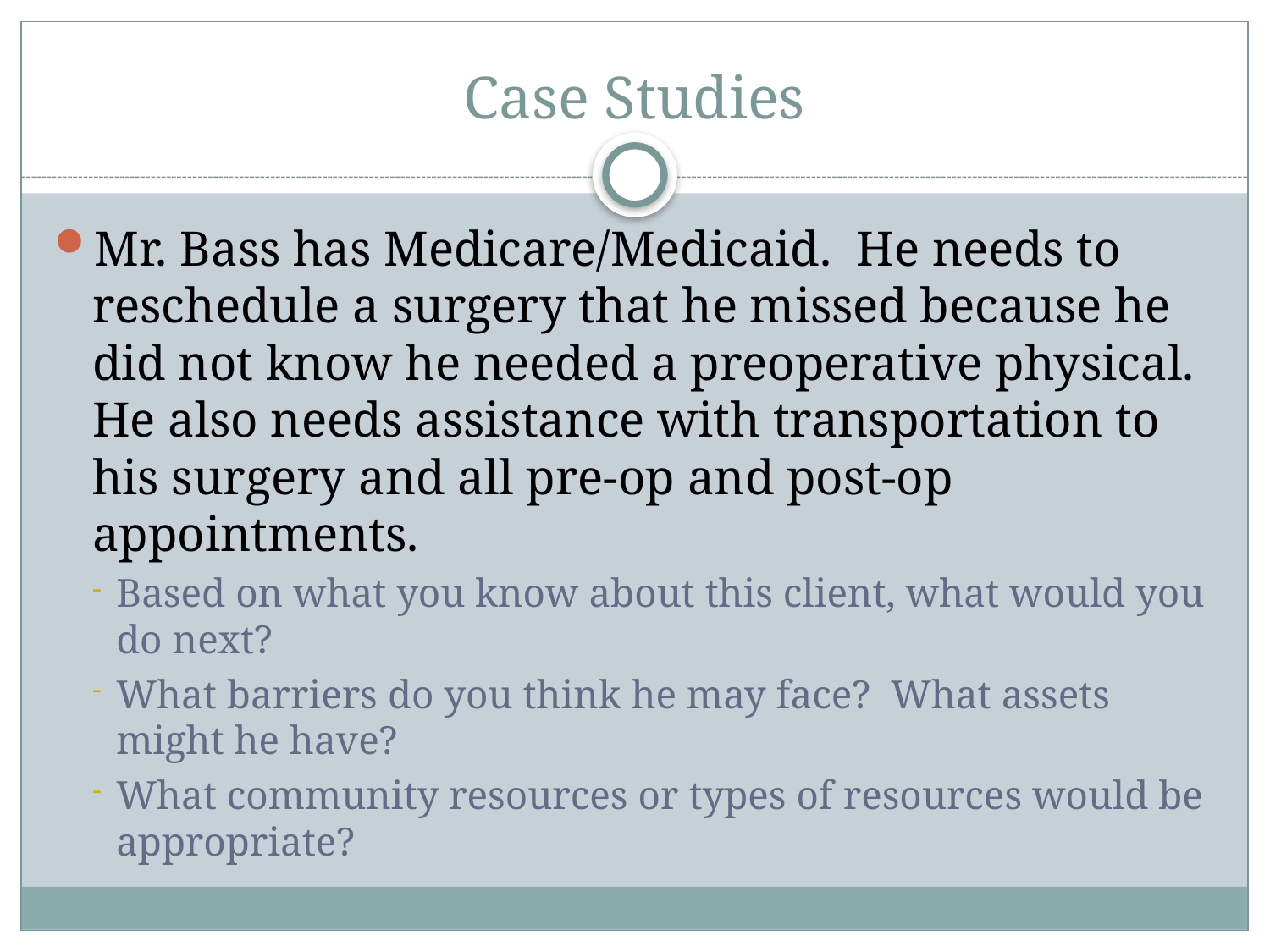

# Case Studies
Mr. Bass has Medicare/Medicaid. He needs to reschedule a surgery that he missed because he did not know he needed a preoperative physical. He also needs assistance with transportation to his surgery and all pre-op and post-op appointments.
Based on what you know about this client, what would you do next?
What barriers do you think he may face? What assets might he have?
What community resources or types of resources would be appropriate?

## Slide 40
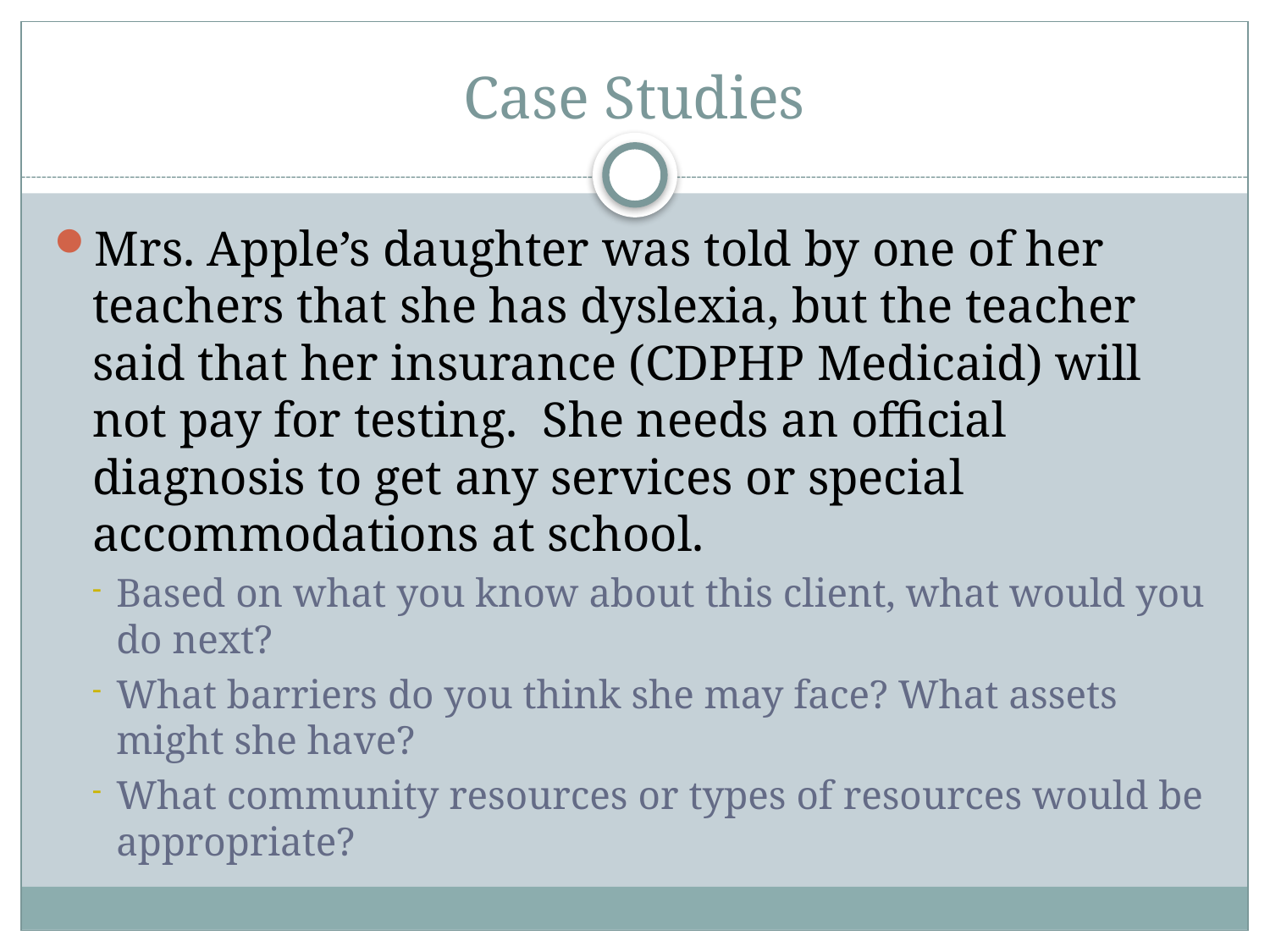

# Case Studies
Mrs. Apple’s daughter was told by one of her teachers that she has dyslexia, but the teacher said that her insurance (CDPHP Medicaid) will not pay for testing. She needs an official diagnosis to get any services or special accommodations at school.
Based on what you know about this client, what would you do next?
What barriers do you think she may face? What assets might she have?
What community resources or types of resources would be appropriate?

## Slide 41
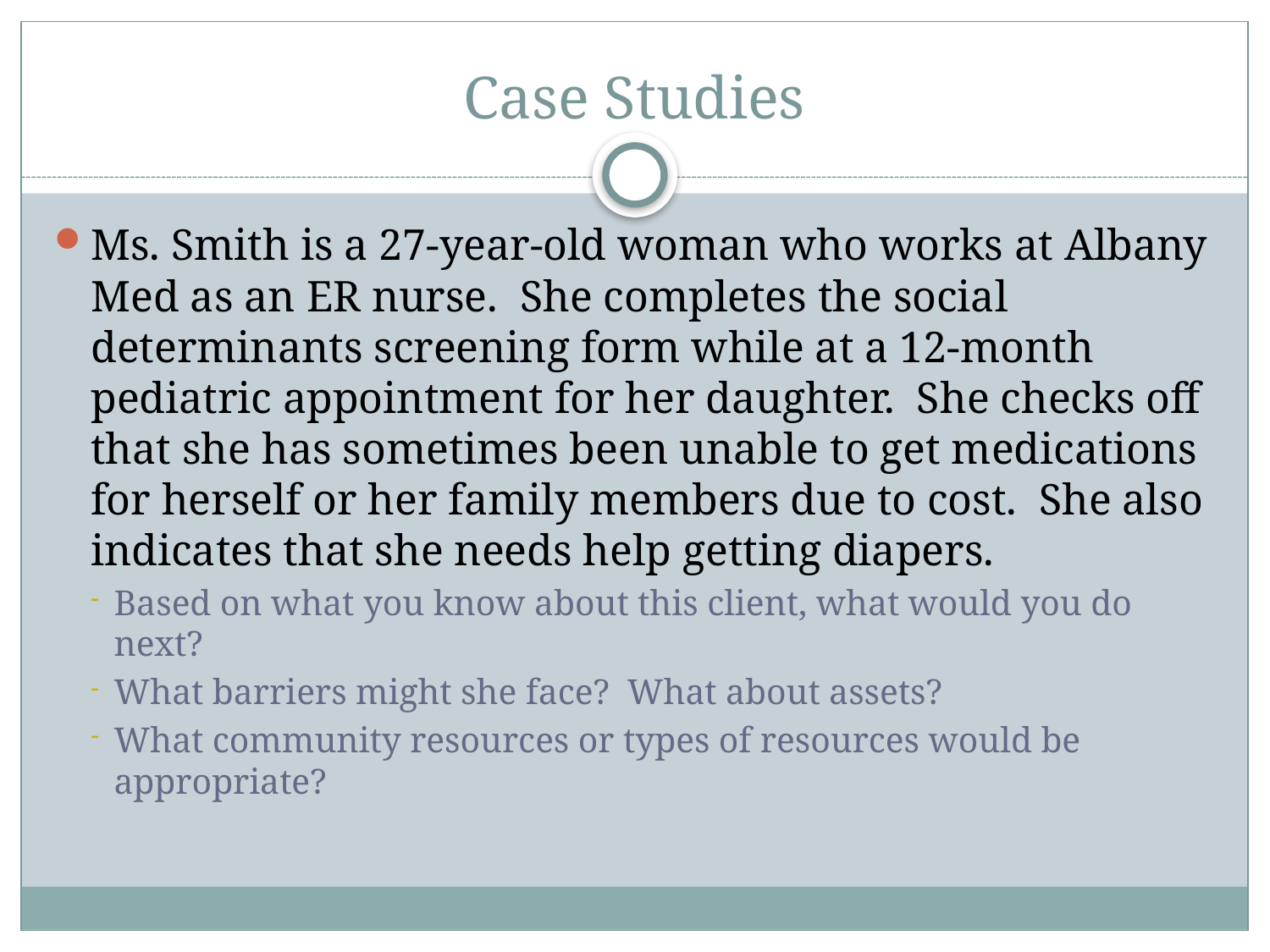

# Case Studies
Ms. Smith is a 27-year-old woman who works at Albany Med as an ER nurse. She completes the social determinants screening form while at a 12-month pediatric appointment for her daughter. She checks off that she has sometimes been unable to get medications for herself or her family members due to cost. She also indicates that she needs help getting diapers.
Based on what you know about this client, what would you do next?
What barriers might she face? What about assets?
What community resources or types of resources would be appropriate?
