# Supplementary material for: A Case-Based Workshop Training Medical Students in Assessing Social Determinants of Health Needs and Connecting With Community Resources
Source: MedEdPORTAL. 2022 Mar 21;18:11232. doi: 10.15766/mep_2374-8265.11232 (PMC8934752; doi:10.15766/mep_2374-8265.11232)
Supplement: Supplementary file 1 — Training Slides.pptxFacilitation Guide.docxSession Evaluation.docxEvaluation Answer Key.docx [file mep_2374-8265.11232-s001.zip › C. Session Evaluation.docx]

**Community resources student training- pre-test**

Please indicate your level of agreement with the below statements:

|  | Strongly Agree | Agree | Neither Agree Nor Disagree | Disagree | Strongly Disagree |
| --- | --- | --- | --- | --- | --- |
| I feel confident that I can assess an individual’s barriers to achieving good health. |  |  |  |  |  |
| I feel confident that I can assess an individual’s barriers accessing the healthcare system. |  |  |  |  |  |
| I am knowledgeable about the community resources available in the Albany area. |  |  |  |  |  |
| I feel confident that I can effectively link individuals with resources in the Albany area. |  |  |  |  |  |
| I can describe the basic eligibility and benefits information for Medicaid. |  |  |  |  |  |
| I can describe the basic eligibility and benefits information for Medicare. |  |  |  |  |  |
| I feel confident in helping individuals effectively utilize their health insurance. |  |  |  |  |  |
| I understand the historical context for mistrust of the healthcare system by marginalized communities. |  |  |  |  |  |
| I can communicate effectively with patients from backgrounds that are significantly different from my own. |  |  |  |  |  |

Knowledge-based Questions

What phone number can you call for help finding community resources?

- 511
- 211
- 311
- 911

Medicaid recipients are eligible for transportation assistance to medical appointments.

- True
- False

If a patient in Albany needed assistance getting diapers, who would you call?

- Salvation Army
- Food Pantries for the Capital District
- American Red Cross
- Albany City School District

Medicaid insurance is available for (check all that apply):

- Individuals who are low income.
- Individuals who are 65 or older.
- Individuals who have been receiving Social Security Disability Insurance for at least two years.
- Individuals of any age or income who chose to buy into the program.

Medicare insurance is available for (check all that apply):

- Individuals who are low income.
- Individuals who are 65 or older.
- Individuals who have been receiving Social Security Disability Insurance for at least two years.
- Individuals of any age or income who chose to buy into the program.

Can someone have Medicare and Medicaid at the same time?

- Yes
- No

True or False: The men who participated in the Tuskegee Syphilis Study were given the benefit of proper informed consent before enrollment

A 2001 Commonwealth Fund Survey revealed which trends regarding care experiences amongst people of color (check all that apply):

- Minorities face greater difficulty in communicating with physicians
- Hispanics and African Americans are more likely to feel treated with disrespect
- Minorities feel they are less involved in their healthcare decisions than they would like to be
- Minorities believe they would receive better healthcare if they were of a different race and/or ethnicity

**Community resources student training- post-test**

After having gone through the training, please indicate your level of agreement with the below statements:

|  | Strongly Agree | Agree | Neither Agree Nor Disagree | Disagree | Strongly Disagree |
| --- | --- | --- | --- | --- | --- |
| I feel confident that I can assess an individual’s barriers to achieving good health. |  |  |  |  |  |
| I feel confident that I can assess an individual’s barriers accessing the healthcare system. |  |  |  |  |  |
| I am knowledgeable about the community resources available in the Albany area. |  |  |  |  |  |
| I feel confident that I can effectively link individuals with resources in the Albany area. |  |  |  |  |  |
| I can describe the basic eligibility and benefits information for Medicaid. |  |  |  |  |  |
| I can describe the basic eligibility and benefits information for Medicare. |  |  |  |  |  |
| I feel confident in helping individuals effectively utilize their health insurance. |  |  |  |  |  |
| I understand the historical context for mistrust of the healthcare system by marginalized communities. |  |  |  |  |  |
| I can communicate effectively with patients from backgrounds that are significantly different from my own. |  |  |  |  |  |

What phone number can you call for help finding community resources?

- 511
- 211
- 311
- 911

True or false: Medicaid recipients are eligible for transportation assistance to medical appointments.

If a patient in Albany needed assistance getting diapers, who would you call?

- Salvation Army
- Food Pantries for the Capital District
- American Red Cross
- Albany City School District

Medicaid insurance is available for (check all that apply):

- Individuals who are low income.
- Individuals who are 65 or older.
- Individuals who have been receiving Social Security Disability Insurance for at least two years.
- Individuals of any age or income who chose to buy into the program.

Medicare insurance is available for (check all that apply):

- Individuals who are low income.
- Individuals who are 65 or older.
- Individuals who have been receiving Social Security Disability Insurance for at least two years.
- Individuals of any age or income who chose to buy into the program.

Can someone have Medicare and Medicaid at the same time? Yes/No

True or False: The men who participated in the Tuskegee Syphilis Study were given the benefit of proper informed consent before enrollment

The 2001 Commonwealth Fund Survey revealed which trends regarding care experiences amongst people of color (check all that apply):

- Minorities face greater difficulty in communicating with physicians
- Hispanics and African Americans are more likely to feel treated with disrespect
- Minorities feel they are less involved in their healthcare decisions than they would like to be
- Minorities believe they would receive better healthcare if they were of a different race and/or ethnicity

Did you gain new knowledge from this training? Yes, no, somewhat

Please describe: ________________________________________________________________

Do you see yourself using this information during service learning? Yes, no, not sure

Do you see yourself using this information in your future medical practice? Yes, no, not sure

Is there anything else you would have liked to learn more about?
