# Supplementary material for: A Case-Based Workshop Training Medical Students in Assessing Social Determinants of Health Needs and Connecting With Community Resources
Source: MedEdPORTAL. 2022 Mar 21;18:11232. doi: 10.15766/mep_2374-8265.11232 (PMC8934752; doi:10.15766/mep_2374-8265.11232)
Supplement: Supplementary file 1 — Training Slides.pptxFacilitation Guide.docxSession Evaluation.docxEvaluation Answer Key.docx [file mep_2374-8265.11232-s001.zip › D. Evaluation Answer Key.docx]

**Community resource student training evaluation: Answer key**

**The correct answers are indicated with the asterisk.**

1. What phone number can you call for help finding community resources?

- 511
- 211*
- 311
- 911

Explanation: 211 is a phone referral line through United Way that provides information and referral services to local human services agencies. It is a useful resource to use if you are unsure of where to start to find a specific resource for a client.

1. Medicaid recipients are eligible for transportation assistance to medical appointments.

- True*
- False

Explanation: Medicaid offers free bus passes or a free taxi ride (if a medical reason certified by a doctor does not allow you to take public transportation) for all their enrollees. Details may vary by state.

1. If a patient in Albany [substitute Albany for your own city] needed assistance getting diapers, who would you call?

- Salvation Army
- Food Pantries for the Capital District*
- American Red Cross
- Albany City School District

Explanation: This organization is the local National Diaper Bank supplier in our area, to find your own supplier you can call the 211 hotline or check the National Diaper Bank Network [member directory](https://nationaldiaperbanknetwork.org/member-directory/). (URL is optional- can be used to help find diaper bank provider in your area)

1. Medicaid insurance is available for (check all that apply):

- Individuals who are low income *
- Individuals who are 65 or older
- Individuals who have been receiving Social Security Disability Insurance for at least two years
- Individuals of any age or income who chose to buy into the program

Explanation: Medicaid provides health insurance specifically only to low-income individuals/families.

1. Medicare insurance is available for (check all that apply):

- Individuals who are low income.
- Individuals who are 65 or older. *
- Individuals who have been receiving Social Security Disability Insurance for at least two years. *
- Individuals of any age or income who chose to buy into the program.

Explanation: Medicare provides health insurance specifically only for individuals over the age of 65 or anyone receiving SSDI for two or more years.

1. Can someone have Medicare and Medicaid at the same time?

- Yes*
- No

Explanation: You can be dual-eligible for both Medicare and Medicaid if you are over the age of 65/ been receiving SSDI for two years and are low income.

1. The men who participated in the Tuskegee Syphilis Study were given the benefit of proper informed consent before enrollment.

True

False*

Explanation: The men who participated in this study had Syphilis and were promised proper medical treatment but were denied that treatment (penicillin) for a study to show the natural progression of the disease.

1. A 2001 Commonwealth Fund Survey revealed which trends regarding care experiences amongst people of color (check all that apply):

- Minorities face greater difficulty in communicating with physicians. *
- Hispanics and African Americans are more likely to feel treated with disrespect. *
- Minorities feel they are less involved in their healthcare decisions than they would like to be. *
- Minorities believe they would receive better healthcare if they were of a different race and/or ethnicity. *

Explanation: These are the four figures shown in training slide 32 when discussing medical mistrust.
